# Characterization of lactylation-related subtypes and diagnostic markers in myocardial ischemic reperfusion injury using weighted gene coexpression network analysis and machine learning

**DOI:** 10.3389/fimmu.2026.1761225

**Published:** 2026-04-21

**Authors:** Jie Bai, Yang Lu, Haidong Wei, Kui Wang, Wei Li, Pengbo Zhang

**Affiliations:** Department of Anesthesiology, The Second Affiliated Hospital of Xi’an Jiaotong University, Shaanxi, China

**Keywords:** diagnostic markers, lactylation, machine learning, myocardial ischemia-reperfusion injury, weighted gene coexpression network analysis

## Abstract

**Introduction:**

Myocardial ischemia-reperfusion injury (MIRI) is a secondary injury that occurs after treatment for ischemic heart disease. This study aimed to identify key lactylation-related genes (LRGs) in MIRI to enable early diagnosis and reveal potential therapeutic targets for improved patient outcomes.

**Methods:**

We analyzed MIRI gene expression datasets from the Gene Expression Omnibus database using differential gene expression and weighted gene coexpression network analyses to determine key genes and coexpression modules. LRGs from the GeneCards database were examined to reveal associations with MIRI. Consensus clustering was used to classify MIRI into distinct subtypes, and machine learning models were developed for diagnostic purposes. Immune cell infiltration was evaluated using CIBERSORT. Key findings were validated via western blot, and an *in vitro* hypoxia -reoxygenation model of HL-1 cardiomyocytes was employed to verify gene expression patterns.

**Results:**

We identified seven significantly expressed LRGs in MIRI: *G6pd*, *Tkt*, *Eif2s2*, *Pabpc1*, *Itgb2*, *Cenpf*, and *Runx2*. Four of these genes (*G6pd*, *Itgb2*, *Pabpc1*, and *Runx2*) showed consistent expression in cell-based assays, supporting their biomarker potential. Enrichment analysis revealed that these genes primarily clustered in cellular division, cytoskeletal reorganization, and multiple signaling pathways, highlighting their critical roles in myocardial injury. Notably, lactylation modifications may modulate immune responses and cellular signaling in MIRI, suggesting therapeutic relevance.

**Discussion:**

In summary, this study identified seven robust diagnostic biomarkers for MIRI and demonstrated distinct molecular subtypes, offering key insights into its pathogenesis and providing a foundation for developing early diagnosis and personalized therapeutic strategies.

## 1 Introduction

Ischemic heart disease (IHD) is the leading cause of age-standardized mortality worldwide, accounting for approximately 16% of all deaths annually (1). Despite advances in reperfusion therapies that have reduced mortality rates, these treatments can trigger acute myocardial injury, commonly known as Myocardial ischemia-reperfusion injury (MIRI) (2). This condition involves complex physiological responses, including oxidative stress, inflammation, mitochondrial dysfunction, and calcium overload, all of which exacerbate cardiac damage and increase the risk of arrhythmias and heart dysfunction (3). Studies have shown that 30%–50% of patients experiencing acute myocardial infarction develop MIRI following interventional procedures, which can expand the infarct and raise the risk of heart failure by 2.1-fold within a year (4). Current diagnostic tools, such as electrocardiograms, have limited sensitivity for detecting minor injuries, and high-sensitivity cardiac troponin tests often yield false negatives in the early stages of injury, restricting their clinical utility (5). Moreover, interventions aimed at restoring blood flow and reducing oxidative stress only decrease heart injury by approximately 20%, frequently failing to prevent cellular damage or improve long-term health outcomes (6). Although bioinformatics-based approaches have shown potential, the lack of specific and sensitive biomarkers for MIRI underscores an urgent need for new diagnostic and therapeutic targets to improve early detection and disease management.

Lactylation is a newly identified post-translational modification that involves adding lactate to lysine residues on proteins, thereby modulating their function and intracellular signaling pathways (7). This process is primarily driven by elevated lactate levels under hypoxic conditions, serving not only as a marker of metabolic activity but also as a regulator of gene expression and inflammation during ischemic events (8). Evidence from cancer biology and other disease models indicates that lactylation dynamically influences metabolic and immune pathways, linking metabolic shifts to adverse health outcomes (9, 10). In MIRI, characterized by substantial lactate accumulation, lactylation may modify protein function and exacerbate tissue damage. However, its precise role in cardiovascular disease, especially MIRI, remains poorly understood. Investigating lactylation-related genes (LRGs) in MIRI may reveal novel biomarkers and therapeutic targets.

**Figure 1:**
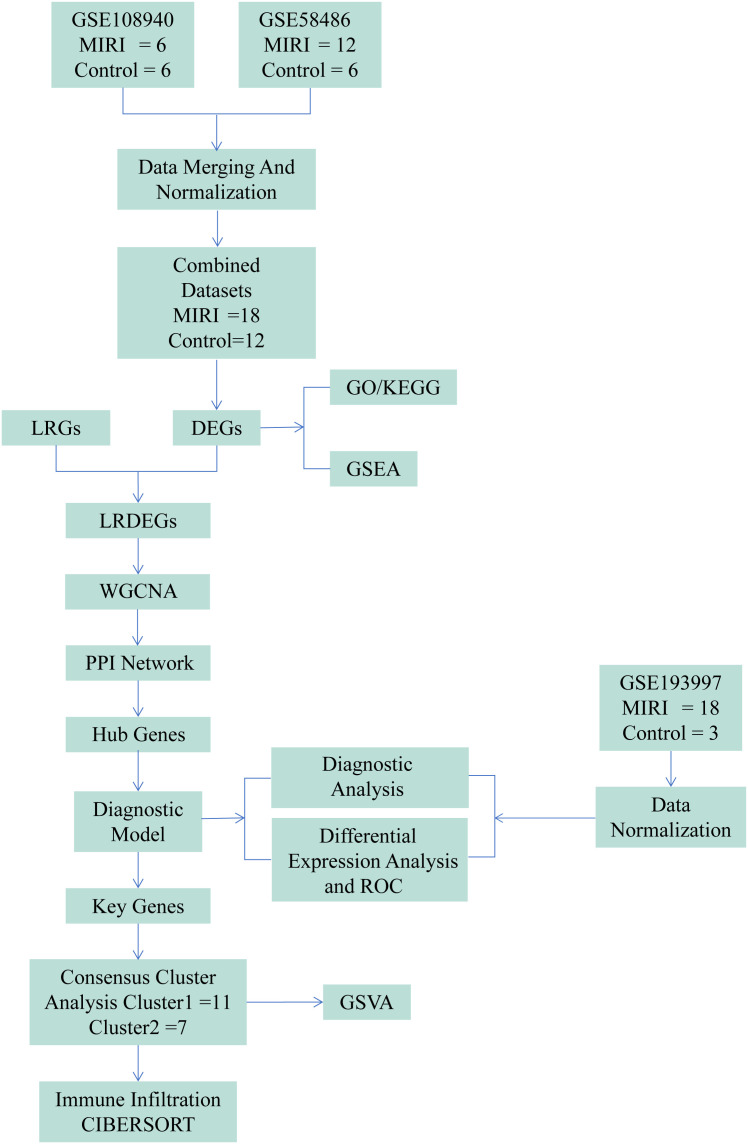
Flow chart for the comprehensive analysis of LRDEGs. MIRI, Myocardial Ischemic Reperfusion Injury; GSEA, Gene Set Enrichment Analysis; DEGs, Differentially Expressed Genes; LRGs, Lactyling-related Genes; GO, Gene Ontology; KEGG, Kyoto Encyclopedia of Genes and Genomes; ROC, Receiver Operating Characteristic; LRDEGs, Lactyling-related Differentially Expressed Genes; GSVA, Gene Set Variation Analysis; WGCNA, Weighted Correlation Network Analysis, PPI Network, Protein-protein Interaction Network.

This study aimed to identify key LRGs in MIRI, assess their functional roles and regulatory networks, construct a diagnostic model, and explore immune characteristics across lactylation-based MIRI subtypes. We also validated the expression of key LRGs through *in vitro* experiments, providing a foundation for early diagnosis and targeted therapy. By integrating transcriptomic data from MIRI mouse models with human LRGs from GeneCards, we identified conserved LRGs and associated molecular pathways. Machine learning algorithms further identified seven key genes that were used to construct a diagnostic model. Furthermore, consensus clustering highlighted two distinct MIRI subtypes. Moreover, key findings were validated in HL-1 cardiomyocytes under oxygen and glucose deprivation. The overall methodological framework employed in this study is illustrated in **Figure 1**. This research lays the groundwork for advancing diagnostic and targeted therapeutic strategies for MIRI.

## 2 Materials and methods

### 2.1 Data collection and initial processing

MIRI datasets GSE108940, GSE58486 (11), and GSE193997 (12) were downloaded from the Gene Expression Omnibus (GEO) database (13) using the R package GEOquery (v2.70.0) (14). All samples were derived from *Mus musculus* heart tissue. The microarray platforms included GPL7202 (GSE108940), GPL18802 (GSE58486), and GPL17021 (GSE193997). Datasets GSE108940 and GSE58486 were merged after batch effect correction to form a training cohort, which was used for feature selection and model development. Dataset GSE193997, which was not involved in the model building process, served as an independent external validation cohort to assess the model’s generalizability and predictive performance. Detailed sample information is provided in **Table 1**. In particular, GSE108940 included 6 MIRI group samples and 6 sham-operated control samples, GSE58486 contained 12 MIRI group samples and 6 controls, and GSE193997 comprised 18 MIRI and 3 controls. All samples were retained for subsequent analyses.

**Table 1:** GEO microarray chip information.

	GSE108940	GSE58486	GSE193997
Platform	GPL7202	GPL18802	GPL17021
Species	Mus musculus	Mus musculus	Mus musculus
Tissue	heart tissue	heart tissue	heart tissue
Samples in MIRI group	6	12	18
Samples in Control group	6	6	3
Reference	–	PMID:25193973	PMID: 35843476

GEO, Gene Expression Omnibus; MIRI, Myocardial Ischemic Reperfusion Injury.

LRGs were initially retrieved from the GeneCards database (15) using the keyword “lactylation,” retaining protein-coding genes only. Additional LRGs were identified via systematic PubMed literature mining (16). Given that the transcriptomic data were derived from Mus musculus heart tissue, while the LRGs were initially based on human databases and literature, to ensure species consistency, human LRGs were converted into mouse orthologs before subsequent analyses. Specifically, after merging and removing duplicates, 1,251 human LRGs were collected and converted into mouse orthologs using the R package homologene (v1.4.68.19.3.27), resulting in 1,098 mouse LRGs (**Supplementary Table 1**). All downstream analyses, including the intersection with differentially expressed genes (DEGs), were conducted based on the converted mouse gene list, thus ensuring that all analyses were performed within a unified mouse gene framework.

The GSE108940 and GSE58486 datasets were harmonized, and batch effects were corrected using the R package sva (v3.50.0) (17), producing an integrated dataset with 18 MIRI and 12 controls samples. Normalization and annotation were performed using the limma package (v3.58.1) (18). The success of these preprocessing steps was evaluated through distribution boxplots and principal component analysis(PCA) (19).

### 2.2 Identification of lactylation-related differentially expressed genesin myocardial ischemic reperfusion injury

Samples from the integrated dataset were stratified into MIRI and control groups. Differential gene expression analysis was conducted using limma (18), with differentially expressed genes (DEGs) defined by log_2_ fold-change (|log_2_FC|) > 0.5 and adjusted p < 0.05. Volcano plots were generated using the ggplot2 package (v3.4.4) to visualize DEGs.

LRDEGs were defined by intersecting DEGs with mouse LRGs, with overlaps illustrated in a Venn diagram. Expression profiles of the top 20 LRDEGs were visualized using heatmaps generated via the pheatmap package (v1.0.12).

### 2.3 Functional annotation analysis

Functional annotation of LRDEGs was performed via Gene Ontology (GO) (20) and Kyoto Encyclopedia of Genes and Genomes (KEGG) (21) pathway analyses implemented in the R package clusterProfiler (v4.10.0) (22). GO terms included biological processes, cellular components, and molecular functions. Significance was defined by adjusted p < 0.05 and false discovery rate (FDR) < 0.25, with multiple testing corrected using the Benjamini–Hochberg method.

### 2.4 Gene set enrichment evaluation

Gene set enrichment analysis (GSEA) (23) was used to evaluate the coordinated behavior of predefined gene sets in phenotype-specific expression patterns. Genes from the integrated dataset were ranked using log_2_FC values. GSEA was conducted using clusterProfiler (22) with the Molecular Signatures Database (MSigDB) “m2” gene collection (release 2024.1.Mm.symbols.gmt) (24). Key parameters included a random seed initialization of 2022, 1000 permutations, and gene set sizes of 10–500 members. Significance thresholds were adjusted p < 0.05 and FDR < 0.25, with multiple comparison correction via the Benjamini–Hochberg method.

### 2.5 Weighted gene coexpression network analysis

WGCNA (25) was applied to identify coexpressed gene modules and their associations with MIRI phenotypes, using the R package WGCNA (v1.72-5) (26). Pairwise correlations among genes were calculated and transformed into a weighted adjacency matrix to construct a scale-free coexpression network. Genes were clustered hierarchically into modules, each represented by a distinct color. Module significance was determined based on correlations with clinical traits to identify biologically relevant modules.

The top 20% most variable genes from the integrated GEO dataset were selected for network construction. Key parameters were minimum module size = 50, soft-threshold power = 18, scale-free topology fit index = 0.8, module merge cut height = 0.2, and minimum module distance = 0.2. Module eigengene analysis identified the candidate module most correlated with MIRI phenotypes (|r| = 0.85), which was prioritized for further investigation. Genes from this module were intersected with LRDEGs in a Venn diagram to identify module-specific candidate genes.

### 2.6 Protein–protein interaction network and hub gene identification

PPI networks are central to biological processes, such as signal transduction, gene regulation, and metabolism. Investigating these networks is essential to understand protein function in MIRI pathogenesis. A PPI network was constructed from the identified module genes using the STRING database (27), with a minimum interaction score of 0.4 (medium confidence).

Highly interconnected regions of the PPI network likely represent functional protein complexes. Hub genes were defined as nodes with the highest connectivity, interacting extensively with other genes. These hub genes were included in subsequent functional analyses to elucidate their roles in MIRI pathogenesis.

### 2.7 Myocardial ischemic reperfusion injury diagnostic model construction

Logistic regression analysis was applied to hub genes to build a diagnostic model for MIRI, with the binary outcome defined as MIRI vs. control. Genes with p < 0.05 were included in the final model, and their expression levels with effect sizes were visualized in a forest plot. Support vector machine recursive feature elimination (SVM-RFE) (28) was applied via the R package e1071 (v1.7-14) to refine feature selection from hub genes in the logistic model, recursively removing features contributing least to classification performance.

Finally, LASSO regression was implemented using the R package glmnet (v4.1-8) (29) under a binomial family, with set seed (500) employed for reproducibility. L1 regularization (λ × |β|) was applied to reduce overfitting and improve model generalizability. Results were visualized through coefficient trajectories and cross-validation plots. Genes with nonzero coefficients in the final model were designated as key genes. A risk score (RiskScore) was calculated based on LASSO coefficients:


$$\text{RiskScore}=\sum [\text{Coefficient }\left(\text{gene i}\right)\times \text{mRNA expression }\left(\text{gene i}\right)]$$

### 2.8 Performance evaluation of the myocardial ischemic reperfusion injury diagnostic model

Diagnostic performance was assessed through receiver operating characteristic (ROC) curve analysis using the pROC package (30) (v1.18.5), calculating area under the curve (AUC) values for the integrated GEO dataset and the external cohort GSE193997. Predictive accuracy was classified as limited (AUC = 0.5–0.7), moderate (0.7–0.9), or excellent (>0.9). A nomogram (31) visualizing key gene contributions was generated via the rms package (v6.7-1), and model calibration was assessed through calibration plots. Clinical utility was assessed using decision curve analysis(DCA) (32) implemented in the ggDCA package (v1.1), determining net benefits across probability thresholds in both datasets.

### 2.9 Verification of differential expression patterns and diagnostic performance

To validate candidate biomarkers, transcriptional profiles were compared between low- and high-risk subgroups in the integrated dataset and GSE193997, with expression differences visualized. The diagnostic potential of each candidate gene was assessed using ROC curve analysis in pROC. AUC values were interpreted as follows: 0.5–0.7, minimal predictive value; 0.7–0.9, moderate diagnostic accuracy; and >0.9, outstanding discrimination. Genes with AUC >0.5 were considered potentially involved in MIRI progression.

### 2.10 Molecular subtype classification of myocardial ischemic reperfusion injury

Consensus clustering, a resampling-based (33) algorithm, was applied to identify molecularly distinct MIRI subtypes and determine the optimal cluster number. Analyses were conducted using ConsensusClusterPlus (34) (v1.62.0) with the expression matrix of key genes from the integrated dataset. Clustering parameters included 2–9 clusters, 80% sample resampling, 1,000 bootstrap iterations, k-means clustering, and Spearman correlation distance. Expression patterns of key genes across subtypes were visualized using hierarchical clustering heatmaps. Intersubtype comparisons were conducted to validate differential gene expression patterns and were presented as box plots.

### 2.11 Gene set variation analysis

GSVA (35) is a nonparametric, unsupervised method that converts gene-level expression into pathway-level enrichment scores, enabling evaluation of pathway activity variations. GSVA was performed using the MSigDB m2 gene set (v2024.1.Mm.symbols.gmt) (14) on the integrated MIRI dataset. Enrichment scores were calculated for all genes to assess functional differences across MIRI subtypes. Significantly enriched pathways were defined by adjusted p < 0.05 with Benjamini–Hochberg correction.

### 2.12 Immune infiltration analysis

CIBERSORT (36) was applied to estimate immune cell subset proportions via linear support vector regression. Analyses were conducted using a mouse-specific immune cell signature, retaining samples with significant immune infiltration (enrichment score > 0). Immune cell compositions were visualized as stacked percentage bar charts.

Interrelationships among immune cell subsets in both MIRI and controls were assessed using Spearman correlation, which were visualized as hierarchical clustering heatmaps using pheatmap. Correlations between key genes and immune cell populations were displayed as bubble plots via ggplot2. Immune infiltration differences between MIRI subtypes (Clusters A and B) were analyzed, and intercorrelations with key genes were visualized as described earlier.

### 2.13 Protein levels of key genes in myocardial ischemic reperfusion injury

HL-1 cardiomyocytes were cultured in Dulbecco’s Modified Eagle Medium containing 10% fetal bovine serum and 1% penicillin–streptomycin. Hypoxia–reoxygenation (H/R) injury was modeled with 4 h of hypoxia (3% O_2_) followed by 24 h of reoxygenation (normoxic conditions). Cell viability was measured using the CCK-8 assay: 1,000 cells/well were seeded in 96-well plates, incubated with CCK-8 reagent for 3 h, and absorbance read at 450 nm.

For Western blotting, proteins were extracted with RIPA buffer, quantified via the BCA assay, separated using SDS-PAGE, transferred to PVDF membranes, and probed with primary antibodies against proteins *G6pd, Tkt, Eif2s2, Pabpc1, Itgb2, Cenpf*, and *Runx2*. Signals were detected using enhanced chemiluminescence.

### 2.14 Statistical analysis

Statistical analyses were conducted in R (v4.3.0). Continuous variables were compared using Student’s *t*-test for normally distributed data or the Mann–Whitney U test for non-normal data in two-group comparisons and the Kruskal–Wallis test in multigroup comparisons. Associations were assessed using Spearman correlation. Statistical significance was defined as two-sided p < 0.05.

## 3 Results

### 3.1 Technology roadmap

Three GEO datasets (GSE108940, GSE58486, GSE193997) were merged after normalization, yielding 18 MIRI and 12 control samples. Differentially expressed genes (DEGs) were identified and intersected with lactate-related genes (LRGs) to define LRDEGs. The top 20 LRDEGs were visualized via heatmap. Functional annotation was performed using GO/KEGG enrichment and GSEA. Weighted gene co-expression network analysis (WGCNA) and protein-protein interaction (PPI) network construction identified hub genes. A diagnostic model was established and evaluated by ROC curves. Finally, consensus clustering classified samples into two subtypes, followed by immune infiltration analysis (CIBERSORT) and GSVA (**Figure 1**).

### 3.2 Standardization of myocardial ischemic reperfusion injury datasets

**Figure 2:**
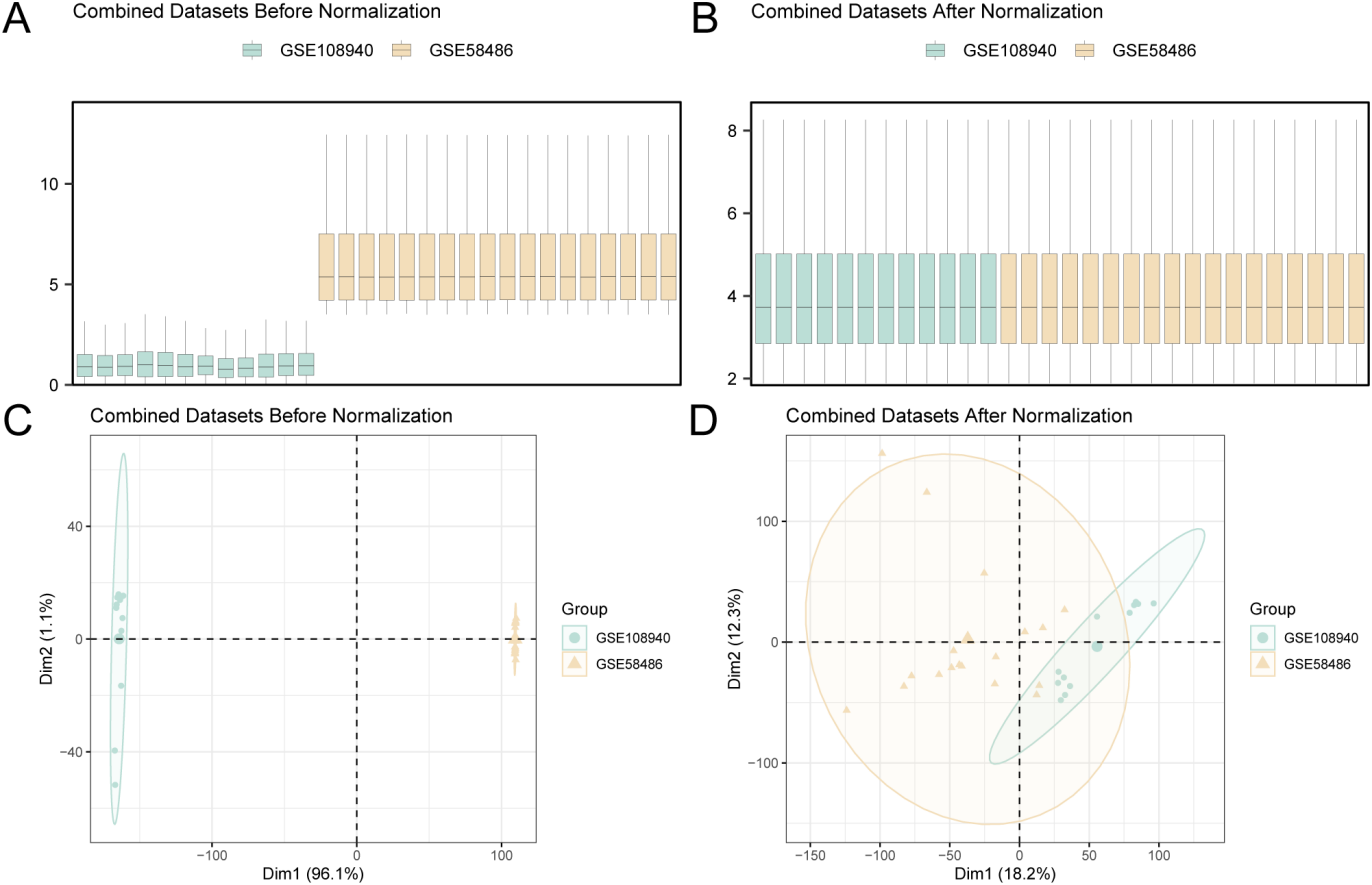
Batch effects removal of MIRIDatasets. **(A)** Box plot of distribution of cardiac ischemia-reperfusion datasets before removing batch effects. **(B)** Post-batch distribution boxplots of GEO Combined Datasets. **(C)** PCA, plot of the cardiac ischemia-reperfusion datasets before debatching. **(D)** PCA plot of Combined GEO Datasets after debatching. PCA, Principal Component Analysis; MIRI, Myocardial Ischemic Reperfusion Injury. Green is the cardiac ischemia-reperfusion (MIRI) dataset GSE108940, and yellow is the cardiac ischemia-reperfusion (MIRI) dataset GSE58486.

Batch effects in the MIRI datasets GSE108940 and GSE58486 were corrected using the sva package, producing an integrated, harmonized GEO dataset. The effectiveness of batch effect correction was determined by evaluating expression distributions via boxplots (**Figures 2A, B**) and assessing low-dimensional embedding similarity through principal component analysis (**Figures 2C, D**). Both approaches confirmed successful mitigation of technical variation in the merged dataset.

### 3.3 Myocardial ischemic reperfusion injury-related degs associated with lactylation

**Figure 3:**
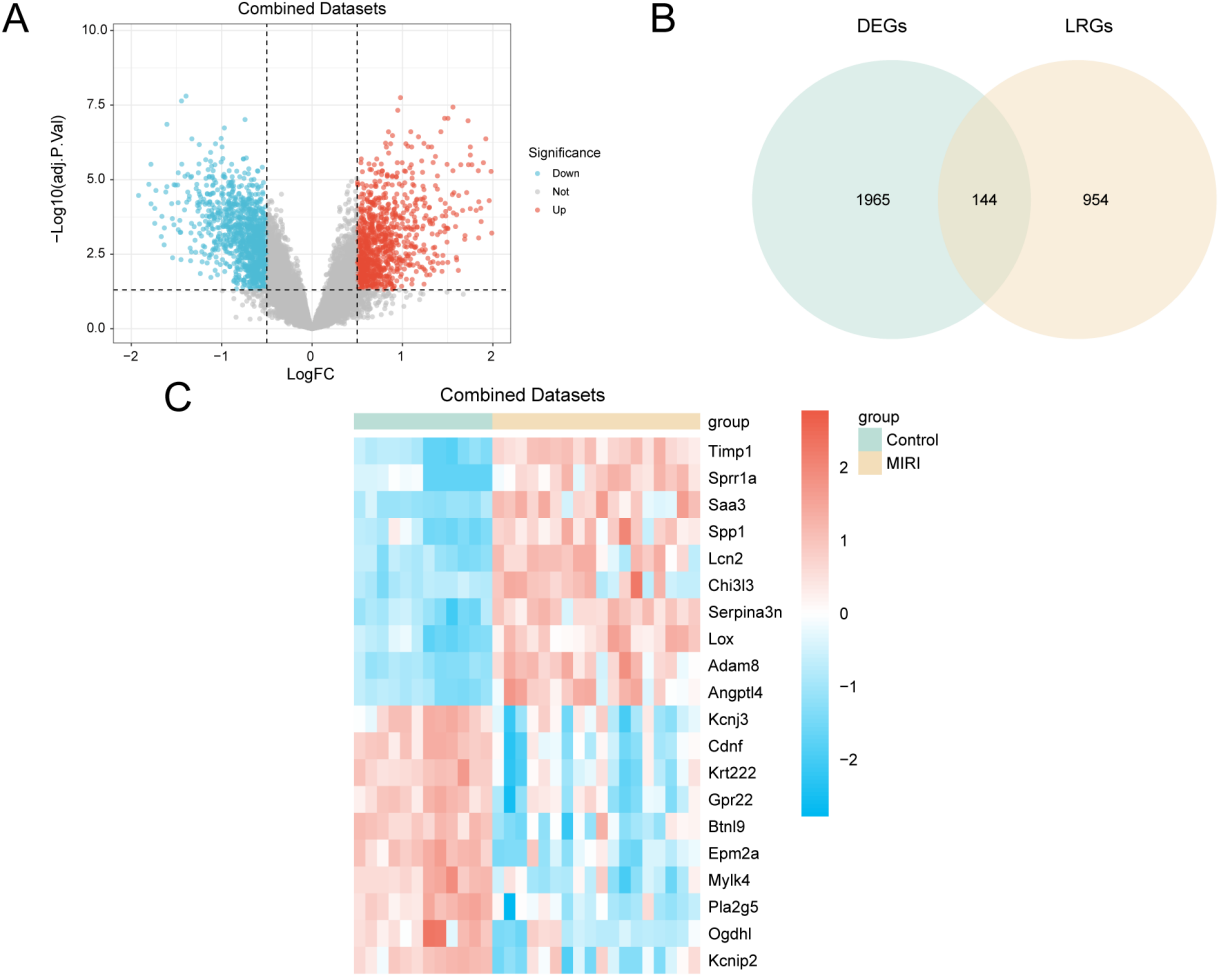
Differential gene expression analysis. **(A)** Volcano plot of differentially expressed genes analysis between cardiac ischemia-reperfusion (MIRI) group and Control (Control) group in Combined GEO Datasets. **(B)** DEGs, Differentially expressed genes and LRGs, lactate modification related genes Venn diagram in the integrated GEO Datasets (Combined Datasets). **(C)** Heat map of differentially expressed genes (LRDEGs) related to lactate modification in the integrated GEO Datasets (Combined Datasets). MIRI, Myocardial Ischemic Reperfusion Injury; DEGs, Differentially Expressed Genes; LRGs, Lactyling-related Genes; LRDEGs, lactyling-related Differentially Expressed Genes. Yellow is the cardiac ischemia-reperfusion (MIRI) group, green is the Control (Control) group. In the heat map, red represents high expression and blue represents low expression.

The integrated GEO dataset was stratified into MIRI and control groups. Differential expression analysis using limma identified 2,109 DEGs under thresholds of |log_2_FC| > 0.5 and adjusted p < 0.05, of which 980 were upregulated and 1,129 downregulated. DEG distribution was visualized in a volcano plot (**Figure 3A**).

Intersecting DEGs with curated LRGs in a Venn diagram (**Figure 3B**) yielded 144 overlapping LRDEGs (**Supplementary Table 2**). A heatmap of the top 20 LRDEGs across sample groups was generated using pheatmap (**Figure 3C**).

### 3.4 GO and KEGG enrichment analyses

Functional enrichment of the 144 LRDEGs in MIRI was assessed through GO and KEGG pathway analyses (**Table 2**), demonstrating substantial enrichment across diverse ontological domains. Within biological processes, LRDEGs were primarily enriched in chromosome segregation processes, including nuclear and sister chromatid separation during mitosis. Cellular component enrichment included chromosomal domains, centromeric regions, condensed chromosomes, actin filaments, and cellular leading edges. Molecular function analysis highlighted enrichment in actin-related binding activities, cytoskeletal structural components, monosaccharide binding, and mRNA 3′-UTR binding interactions.

**Table 2:** Results of GO and KEGG enrichment analysis.

ONTOLOGY	ID	Description	GeneRatio	BgRatio	p value	p.adjust	q value
BP	GO:0098813	nuclear chromosome segregation	20/135	312/28891	3.12E-17	9.08E-14	6.35E-14
BP	GO:0000819	sister chromatid segregation	17/135	218/28891	3.27E-16	4.76E-13	3.33E-13
BP	GO:0007059	chromosome segregation	21/135	416/28891	5.89E-16	5.71E-13	3.99E-13
BP	GO:0000070	mitotic sister chromatid segregation	15/135	177/28891	5.72E-15	4.16E-12	2.91E-12
BP	GO:0140014	mitotic nuclear division	17/135	267/28891	9.57E-15	5.57E-12	3.89E-12
CC	GO:0098687	chromosomal region	15/139	375/28573	5.00E-10	1.01E-07	7.43E-08
CC	GO:0000775	chromosome,centromeric region	13/139	266/28573	6.76E-10	1.01E-07	7.43E-08
CC	GO:0000793	condensed chromosome	14/139	337/28573	1.20E-09	1.20E-07	8.78E-08
CC	GO:0005884	actin filament	9/139	134/28573	2.05E-08	1.54E-06	1.13E-06
CC	GO:0031252	cell leading edge	13/139	415/28573	1.32E-07	7.93E-06	5.82E-06
MF	GO:0003779	actin binding	17/135	448/28407	5.02E-11	2.06E-08	1.66E-08
MF	GO:0051015	actin filament binding	12/135	219/28407	6.55E-10	1.35E-07	1.09E-07
MF	GO:0005200	structural constituent of cytoskeleton	7/135	73/28407	5.87E-08	8.05E-06	6.49E-06
MF	GO:0048029	monosaccharide binding	6/135	92/28407	5.25E-06	0.000539886	0.00043556
MF	GO:0003730	mRNA 3'-UTR binding	6/135	119/28407	2.30E-05	0.00189282	0.001527054
KEGG	mmu05132	Salmonella infection - Mus musculus (house mouse)	12/88	252/9577	2.95E-06	0.000546315	0.000472489
KEGG	mmu04666	Fc gamma R-mediated phagocytosis - Mus musculus (house mouse)	7/88	94/9577	2.33E-05	0.001413455	0.001222448
KEGG	mmu04530	Tight junction - Mus musculus (house mouse)	9/88	170/9577	2.43E-05	0.001413455	0.001222448
KEGG	mmu05135	Yersinia infection - Mus musculus (house mouse)	8/88	136/9577	3.33E-05	0.001413455	0.001222448
KEGG	mmu05230	Central carbon metabolism in cancer - Mus musculus (house mouse)	6/88	69/9577	3.82E-05	0.001413455	0.001222448

GO, Gene Ontology; BP, Biological Process; CC, Cellular Component; MF, Molecular Function; KEGG, Kyoto Encyclopedia of Genes and Genomes.

**Figure 4:**
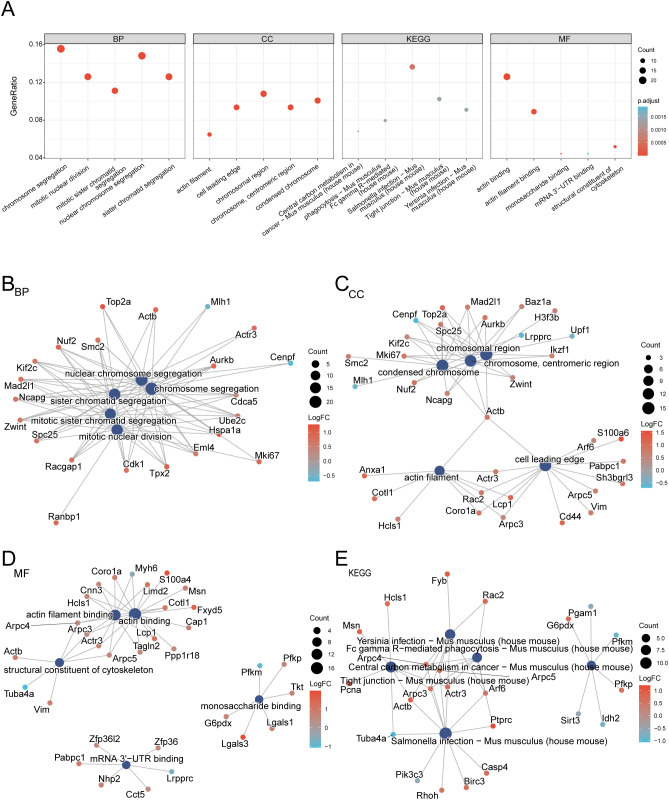
GO enrichment analysis for LRDEGs. **(A)** Bubble map of GO, gene ontology enrichment analysis results of LRGs, lactation-related genes showed; BP, biological process; CC, cellular component; MF, molecular function and biological pathway (KEGG). GO terms and KEGG terms are shown on the abscissa. **(B-D)**. Gene ontology **(GO)** enrichment analysis results of differentially expressed genes (LRDEGs) related to lactate modification network diagram showing: BP **(B)**, CC **(C)**, MF **(D)** and KEGG. **(E)** The dark blue nodes represent entries, and the lines represent the relationship between entries and molecules. In the molecular nodes, red represents up-regulation, and blue represents down-regulation. LRDEGs, Lactyling-related Differentially Expressed Genes; GO, Gene Ontology; KEGG, Kyoto Encyclopedia of Genes and Genomes; BP, Biological Process; CC, Cellular Component; MF, Molecular Function. The bubble size in the bubble plot represents the number of genes, and the color of the bubble represents the size of the adj. P-value, the redder the color, the smaller the adj. P-value, and the bluer the color, the larger the adj. P-value. The screening criteria for GO, gene ontology and pathway (KEGG) enrichment analysis were adj.p < 0.05 and FDR value (q value) < 0.25, and the p value correction method was Benjamini-Hochberg (BH).

KEGG pathways markedly enriched among LRDEGs included *Salmonella* infection, Fc gamma R-mediated phagocytosis, tight junction regulation, *Yersinia* infection, and central carbon metabolism in cancer in *M. musculus*. Enrichment results were visualized through bubble plots (**Figure 4A**) and network diagrams depicting relationships among biological processes, cellular components, molecular functions, and KEGG pathways (**Figures 4B–E**), with node sizes representing molecule counts and edges indicating functional associations.

### 3.5 Gene set variation analysis

**Figure 5:**
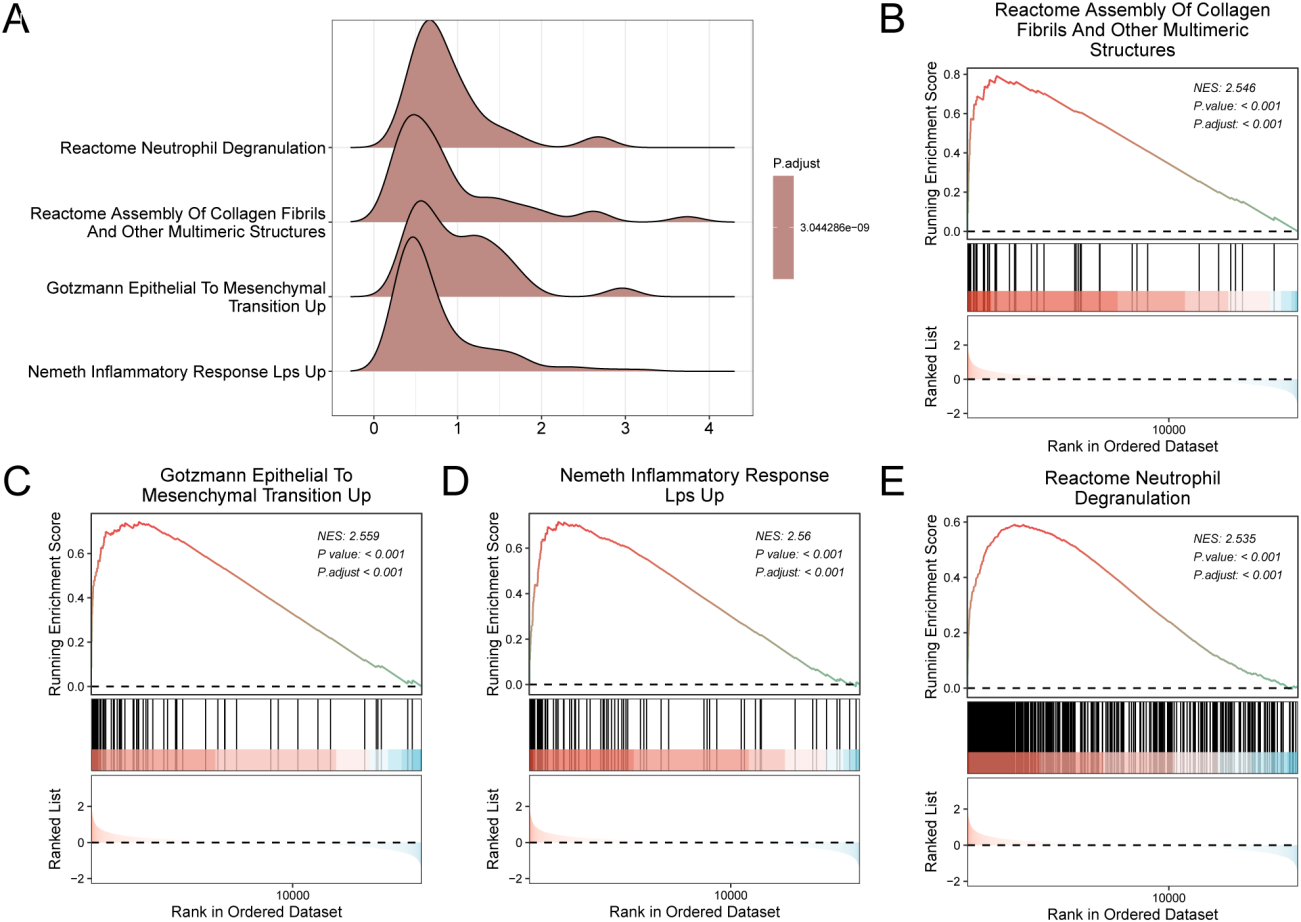
GSEA for combined datasets. **(A)** Gene set enrichment analysis (GSEA) 4 biological functions mountain plot presentation of the cardiac ischemia-reperfusion dataset. **(B–E)**. Gene set enrichment analysis (GSEA) showed that all genes were significantly enriched in REACTOME_ASSEMBLY_OF_COLLAGEN_FIBRILS_AND_OTHER_MULTIMERIC_STRUCTURES **(B)**, GOTZMANN_EPITHELIAL_TO_MESENCHYMAL_TRANSITION_UP **(C)**, NEMETH_INFLAMMATORY_RESPONSE_LPS_UP **(D)**, REACTOME_NEUTROPHIL_DEGRANULATION **(E)**. MIRI, Myocardial Ischemic Reperfusion Injury; GSEA, Gene Set Enrichment Analysis. The screening criteria of gene set enrichment analysis (GSEA) were adj.p < 0.05 and FDR value (q value) < 0.25, and the p value correction method was Benjamini-Hochberg (BH).

GSEA was performed on the integrated GEO dataset to assess genome-wide expression patterns associated with MIRI. This unbiased approach evaluated the involvement of all genes in relevant biological pathways. **Figure 5A** illustrates the relationship between cellular components and their associated molecular functions, with full details provided in **Table 3**. Genes in the MIRI dataset were significantly enriched in the assembly of collagen fibrils and other multimeric structures (**Figure 5B**), epithelial-to-mesenchymal transition (Gotzmann) (**Figure 5C**), LPS-induced inflammatory response (Nemeth) (**Figure 5D**), and neutrophil degranulation (**Figure 5E**).

**Table 3:** Results of GSEA for combined datasets.

ID	Set size	Enrichment score	NES	pvalue	p.adjust	qvalue
NEMETH_INFLAMMATORY_RESPONSE_LPS_UP	87	0.716560636	2.559725155	1.00E-10	3.04E-09	1.99E-09
GOTZMANN_EPITHELIAL_TO_MESENCHYMAL_TRANSITION_UP	69	0.743350549	2.558748347	1.00E-10	3.04E-09	1.99E-09
REACTOME_ASSEMBLY_OF_COLLAGEN_FIBRILS_AND_OTHER_MULTIMERIC_STRUCTURES	50	0.792635976	2.545843157	1.00E-10	3.04E-09	1.99E-09
REACTOME_NEUTROPHIL_DEGRANULATION	451	0.591272715	2.535198225	1.00E-10	3.04E-09	1.99E-09

GSEA, Gene Set Enrichment Analysis.

### 3.6 Weighted gene association network analysis

**Figure 6:**
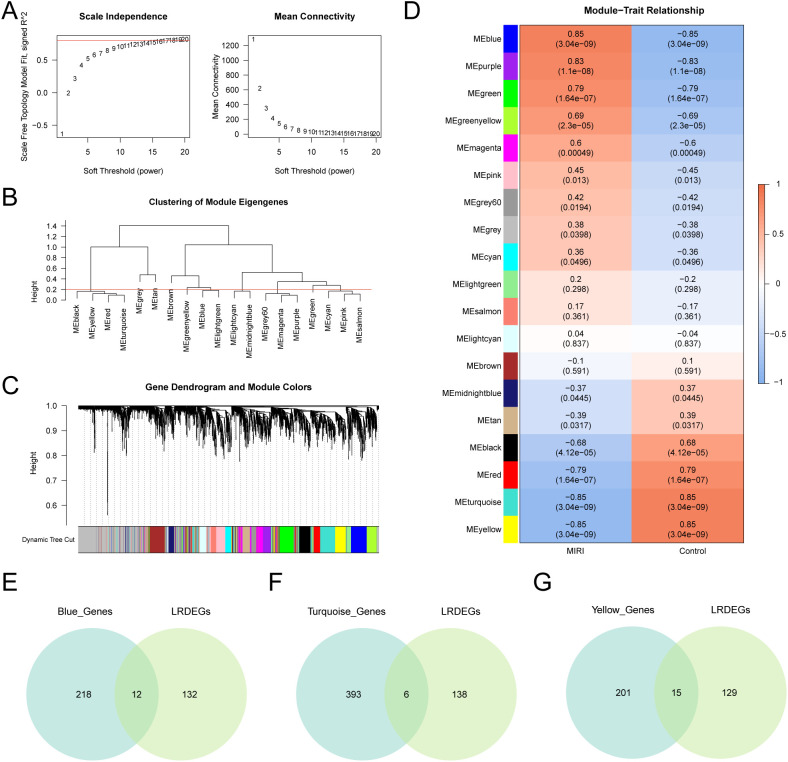
WGCNA for combined datasets. **(A)** Scale-free network presentation of the best soft threshold from weighted gene co-expression network analysis (WGCNA), the left panel shows the best soft threshold and the right panel shows the network connectivity under different soft threshold conditions. **(B)** Display of module clustering results of genes with top 20% variance. **(C)** Presentation of cluster results for genes with top 20% variance. The upper part is divided into hierarchical clustering dendrogram, and the lower part is divided into gene modules. **(D)** The results of correlation analysis between the top 20% of variance gene cluster modules and the MIRI group and the Control (Control) group are shown. **(E, F)**. Venn diagram of 144 differentially expressed genes (LRDEGs) related to lactate modification and the genes contained in the MEblue **(E)**, MEturquoise **(F)**, and MEyellow **(G)** modules. MIRI, Myocardial Ischemic Reperfusion Injury; WGCNA, Weighted Gene Co-Expression Network Analysis; LRDEGs, Lactyling-related Differentially Expressed Genes. The absolute value of correlation coefficient (r value) below 0.3 was weak or no correlation, between 0.3 and 0.5 was weak correlation, between 0.5 and 0.8 was moderate correlation, and above 0.8 was strong correlation. Red represents positive correlation, blue represents negative correlation.

WGCNA was conducted on the top 20% most variable genes from the merged GEO dataset. The scale-free topology fit index was assessed across soft thresholds, selecting an optimal power of 18 to achieve a fit index of 0.8 (**Figure 6A**). A hierarchical clustering tree grouped genes into 19 coexpression modules (**Figure 6B**): MEblue, MEpurple, MEgreen, MEgreenyellow, MEmagenta, MEpink, MEgrey60, MEgrey, MEcyan, MElightgreen, MEsalmon, MElightcyan, MEbrown, MEmidnightblue, MEtan, MEblack, MEred, MEyellow, and MEturquoise. Module–gene associations were visualized (**Figure 6C**), and module eigengene correlations with MIRI or control status were evaluated (**Figure 6D**). Three modules, namely, MEblue, MEturquoise, and MEyellow, showed the highest correlation with MIRI (|r| = 0.85) and were selected for further analysis. Intersecting the genes within these module with the 144 LRDEGs via Venn diagrams yielded 33 candidate module genes (**Figures 6E–G**; **Supplementary Table 3**).

### 3.7 PPI network and hub gene screening

**Figure 7:**
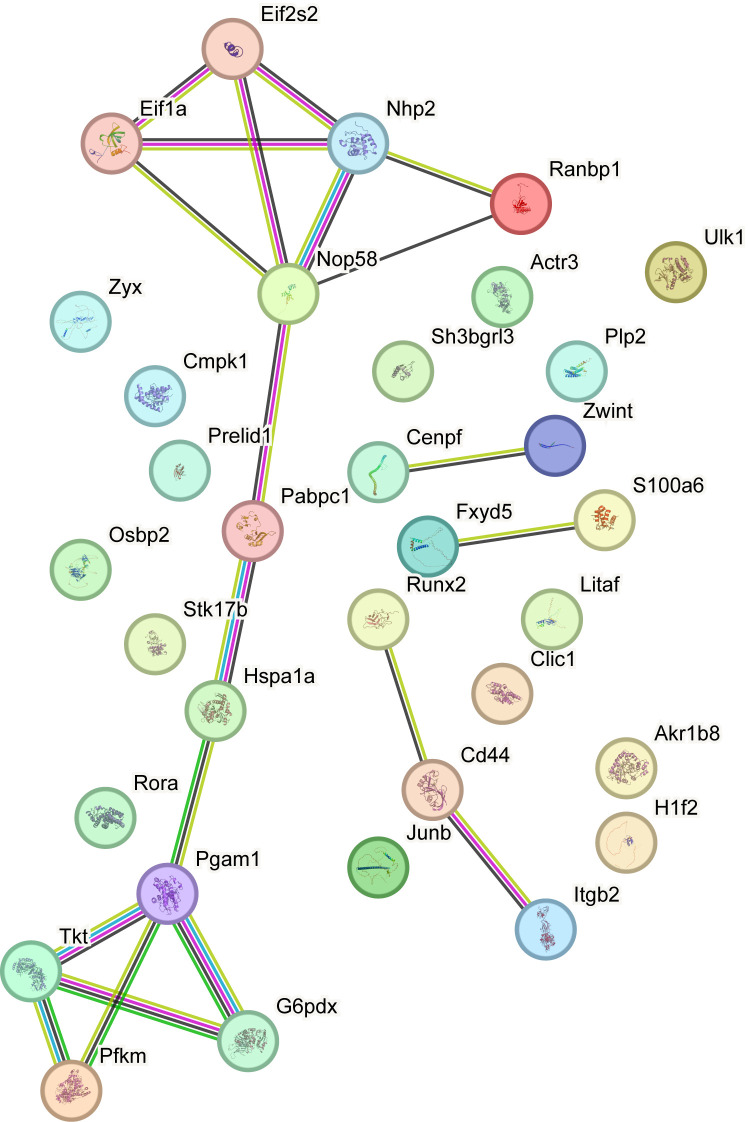
PPI network analysis. Protein-protein interaction Network (PPI Network) of Module Genes calculated by STRING database. PPI Network, Protein-protein Interaction Network.

A PPI network for the 33 module genes was constructed using the STRING database (**Figure 7**). Among these, 18 module genes, namely, *Cd44, Itgb2, Runx2, Cenpf, Zwint, Eif1a, Nhp2, Nop58, Eif2s2, Fxyd5, S100a, G6pd, Tkt, Pgam1, Hspa1a, Pabpc1, Ranbp1*, and *Pfkm*, formed highly interconnected clusters, serving as hub genes for downstream analyses.

### 3.8 Construction of the myocardial ischemic reperfusion injury diagnostic model

**Figure 8:**
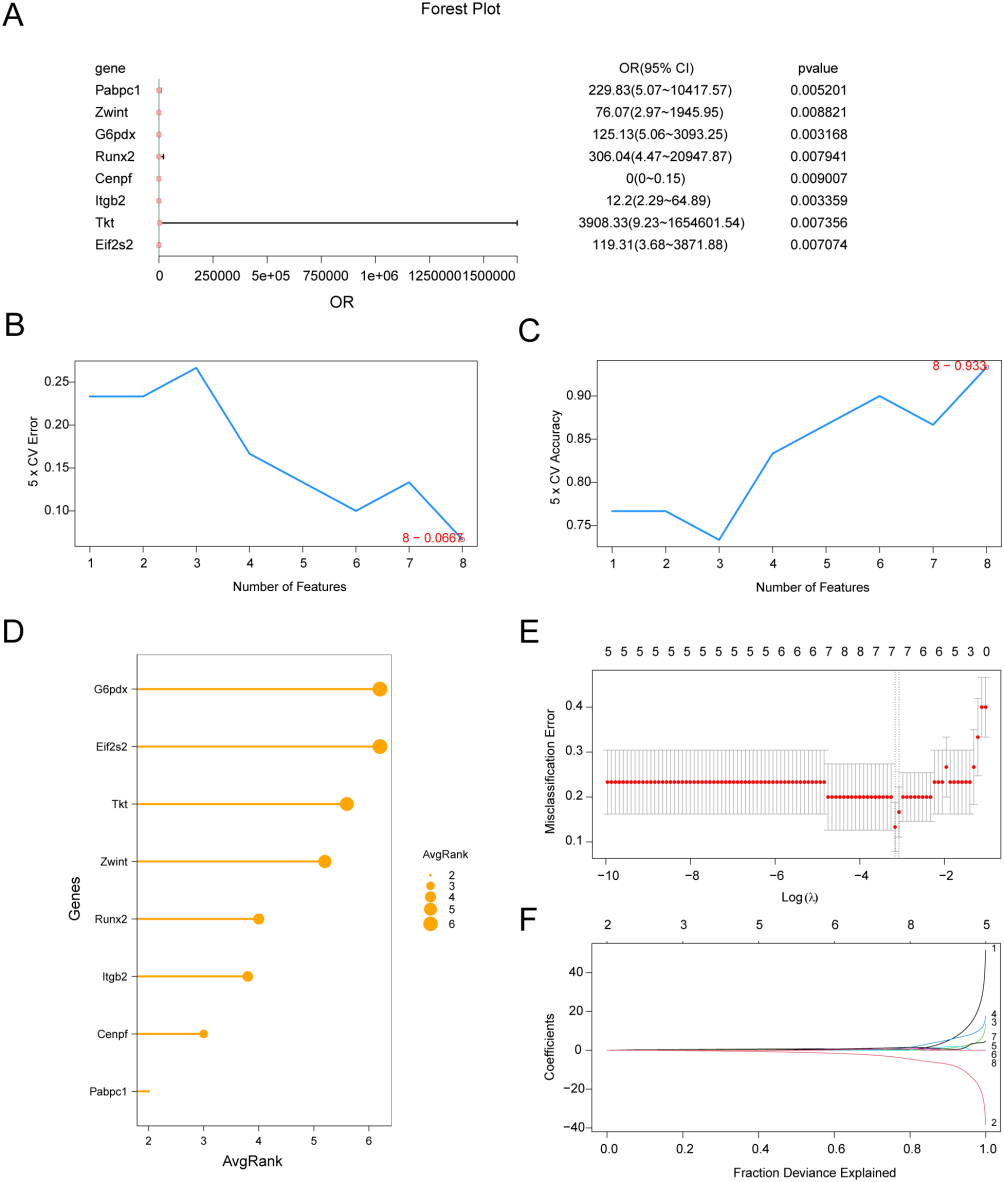
Screening key genes of MIRIBy machine learning. **(A)** Forest Plot of 8 hub genes included in the Logistic regression model in the MIRI diagnostic model. **(B–C)**. The number of genes with the lowest error rate **(B)** and the number of genes with the highest accuracy **(C)** obtained by SVM-RFE algorithm are visualized. **(D)** The average importance ranking lollipop plot of the eight genes with the lowest error rate obtained by the SVM-RFE algorithm. **(E–F)**. Diagnostic model plot **(E)** and variable trajectory plot **(F)** of LASSO regression model. SVM, Support Vector Machine; LASSO, Least Absolute Shrinkage and Selection Operator.


To assess the diagnostic potential of the 18 hub genes, logistic regression was used to construct a predictive model, with results visualized in a forest plot (**Figure 8A**). Eight hub genes, namely*Pabpc1, Zwint, G6pd, Runx2, Cenpf, Itgb2, Tkt*, and *Eif2s2*, were identified as statistically significant predictors (p < 0.01). SVM-RFE with fivefold cross-validation was employed to rank the eight genes, with the eight-gene model achieving minimal error (**Figure 8B**) and maximal accuracy (**Figure 8C**). Thus, the top eight genes by average rank (**Figure 8D**) were further analyzed. LASSO regression was applied to these genes to develop a diagnostic model for MIRI. The LASSO regression model (**Figure 8E**) and variable trajectory (**Figure 8F**) were visualized, resulting in the selection of seven key genes:*G6pd, Tkt, Eif2s2, Pabpc1, Itgb2, Cenpf*, and *Runx2*. Finally, RiskScore was calculated based on LASSO coefficients:

$$\begin{array}{c} RiskScore=Pabpc1\;\times \;\left(0.544\right)\;+\text{ Cenpf }\times \;\left(-3.080\right)+\;Itgb2\\ \;\times \;\left(0.220\right)+\;Runx2\;\times \;\left(0.565\right)+\;Tkt\;\times \;\left(0.626\right)+\\ \;G6pdx\;\times \;\left(1.181\right)+\;Eif2s2\;\times \;\left(0.573\right). \end{array}$$

### 3.9 Internal validation of the myocardial ischemic reperfusion injury diagnostic model

**Figure 9:**
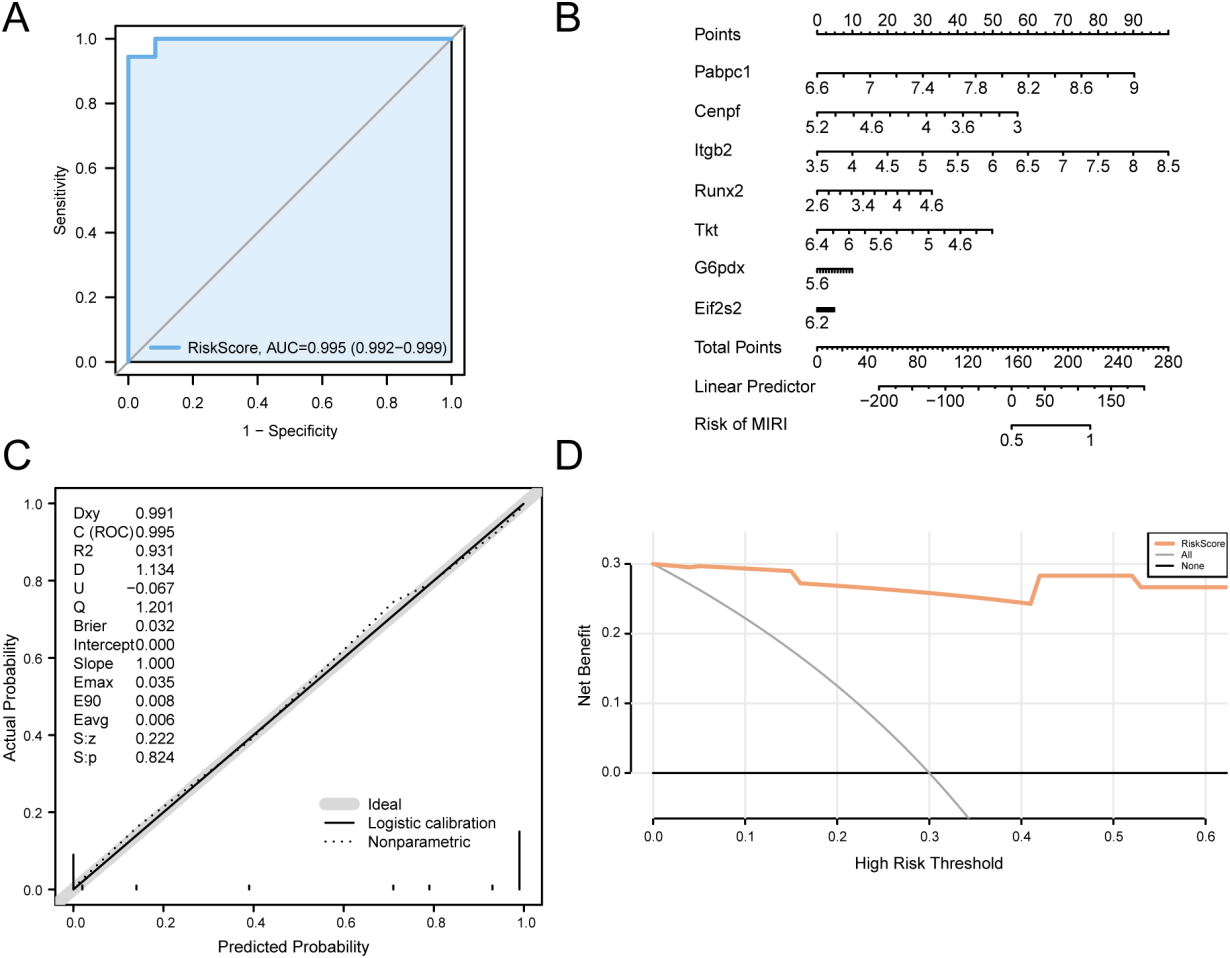
Diagnostic and validation analysis of MIRI. **(A)** ROC curves of logistic regression model in MIRI samples and Control samples. **(B)** Nomograms of Key Genes in Combined GEO Datasets in cardiac ischemia-reperfusion (MIRI) diagnostic models. **(C–D)**. Calibration Curve plot **(C)** and decision curve analysis (DCA) plot **(D)** of Key Genes in Combined Datasets for cardiac ischemia-reperfusion (MIRI) diagnostic model. The ordinate of DCA, decision curve analysis plot is the net benefit, and the abscissa is the Probability Threshold or Threshold Probability. MIRI, Myocardial Ischemic Reperfusion Injury; DCA, Decision Curve Analysis; ROC, Receiver Operating Characteristic; AUC, Area Under the Curve. When AUC > 0.5, it indicates that the expression of the molecule is a trend to promote the occurrence of the event, and the closer the AUC is to 1, the better the diagnostic effect. AUC > 0.9 was associated with high accuracy.

ROC analysis of the RiskScore in the integrated GEO dataset, achieved using pROC, demonstrated the model’s high discriminatory power (AUC > 0.9) for distinguishing MIRI from controls (**Figure 9A**). A nomogram integrating key genes visualized their individual contributions within the combined dataset, with gene *Itgb2* having the highest diagnostic value and gene *Eif2s2* the lowest (**Figure 9B**). Model calibration compared predicted versus observed outcomes (**Figure 9C**), showing minor deviations from the ideal diagonal, indicating acceptable prediction accuracy. Decision curve analysis was employed to examine the diagnostic model’s clinical utility (**Figure 9D**). Across a range of clinically relevant threshold probabilities, the model yielded superior net benefit compared with “treat all” and “treat none” strategies, supporting its potential for clinical translation.

### 3.10 External validation of the myocardial ischemic reperfusion injury diagnostic model

**Figure 10:**
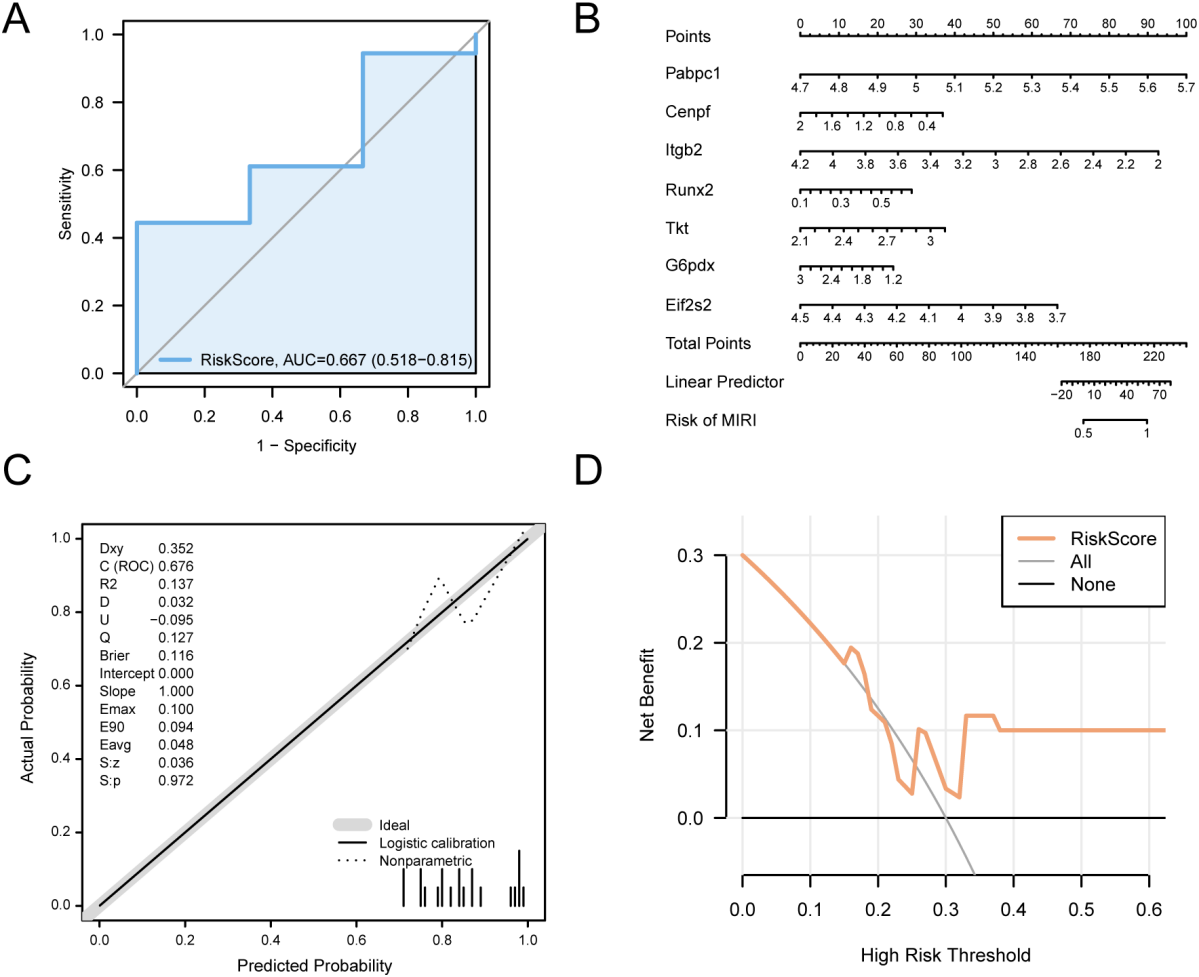
Diagnostic and validation analysis of MIRI. **(A)** ROC curves of logistic regression model in cardiac ischemia-reperfusion (MIRI) samples and Control (Control) samples. **(B)** Nomogram of Key Genes in dataset GSE193997 in cardiac ischemia-reperfusion (MIRI) diagnostic model. **(C, D)**. Calibration Curve plot **(C)** and DCA, decision curve analysis plot **(D)** of the myocadial ischemia-reperfusion (MIRI) diagnostic model based on the Key Genes in dataset GSE193997. The ordinate of DCA, decision curve analysis plot is the net benefit, and the abscissa is the Probability Threshold or Threshold Probability. MIRI, Myocardial Ischemic Reperfusion Injury; DCA, Decision Curve Analysis; ROC, Receiver Operating Characteristic; AUC, Area Under the Curve. When AUC > 0.5, it indicates a diagnostic value better than random chance. Generally, an AUC between 0.7 and 0.9 suggests moderate accuracy, while an AUC > 0.9 indicates high accuracy.

To evaluate the independent predictive ability of the model, dataset GSE193997-which was not involved in the model-building process-was employed as an external validation cohort for verification analysis. To independently validate the diagnostic model, ROC analysis of RiskScore was performed on the GSE193997 dataset via pROC. The model showed limited discriminatory ability (0.5 < AUC < 0.7) for distinguishing MIRI from controls (**Figure 10A**). A nomogram visualized the relative contributions of key genes (**Figure 10B**), with gene *Pabpc1* exhibiting the highest predictive value and gene *G6pd* the lowest. Calibration curves comparing predicted versus observed outcomes (**Figure 10C**) showed minor deviation from the ideal diagonal, indicating slight miscalibration. Decision curve analysis (**Figure 10D**) confirmed that the model provided superior net benefit across a range of threshold probabilities compared with “treat all” or “treat none” strategies, supporting potential clinical applicability despite limited discrimination.

### 3.11 Validation of differential expression between high- and low-risk groups in the integrated GEO dataset

**Figure 11:**
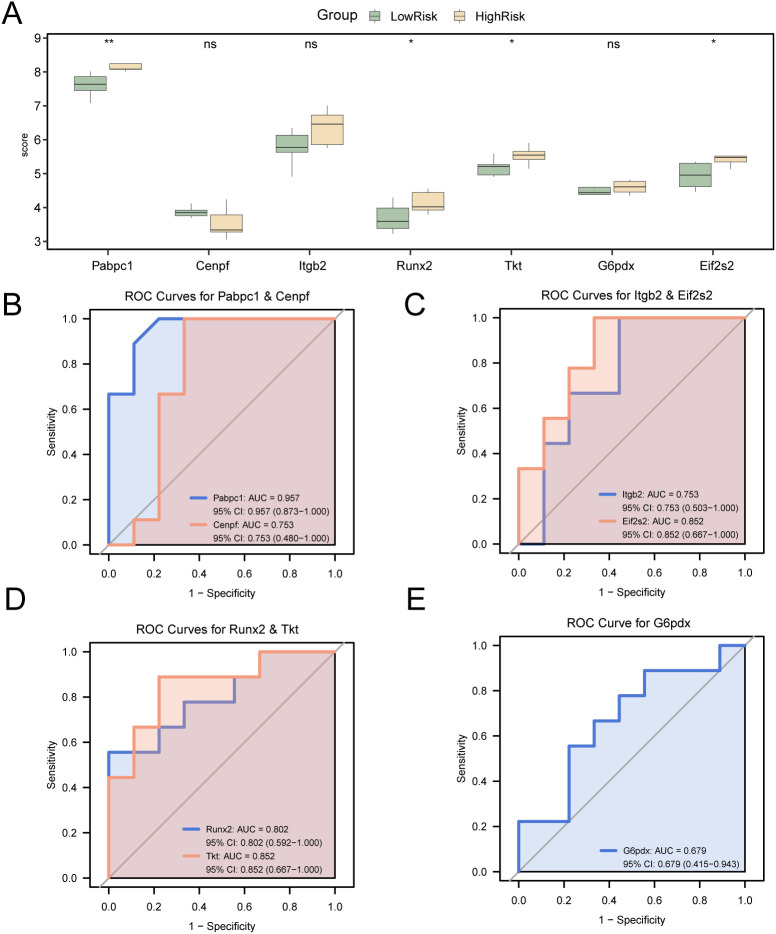
Differential expression validation and ROC curve analysis. **(A)** Group comparison diagram of Key Genes in the LowRisk group and HighRisk group in the cardiac ischemia-reperfusion (MIRI) samples of the Combined GEO Datasets. **(B–G)**. Key Genes Pabpc1 and Cenpf **(B)**, Itgb2 and Runx2 **(C)**, Tkt and G6pd **(D)**, ROC curves of Eif2s2 **(E)** in cardiac ischemia-reperfusion (MIRI) samples from Combined GEO Datasets. ROC, Receiver Operating Characteristic; MIRI, Myocardial Ischemic Reperfusion Injury. ns stands for p value ≥ 0.05, no statistical significance; * represents p value < 0.05, statistically significant; ** represents p value < 0.01 and highly statistically significant. The closer the AUC is to 1, the better the diagnostic performance. When AUC > 0.5, it indicates that the expression of the molecule is a trend to promote the occurrence of the event; AUC between 0.5 and 0.7 had low accuracy, AUC between 0.7 and 0.9 had moderate accuracy, and AUC above 0.9 had high accuracy. In the comparison of groups, green represents the LowRisk group and yellow represents the HighRisk group.

The transcriptional patterns of seven candidate genes (*G6pd, Tkt, Eif2s2, Pabpc1, Itgb2, Cenpf*, and *Runx2*) were examined in the low- and high-risk cohorts from the integrated GEO dataset (**Figure 11A**). Comparative expression profiling revealed significant differences (p < 0.05) in three genes (*Runx2, Tkt*, and *Eif2s2*), with gene *Pabpc1* displaying particularly robust differential expression (p < 0.01). ROC curve analysis was used to evaluate diagnostic potential (**Figures 11B–E**). Gene *G6pd* demonstrated limited predictive value (0.5 < AUC < 0.7); genes *Cenpf, Itgb2, Runx2, Tkt*, and *Eif2s2* exhibited moderate discrimination (0.7 < AUC < 0.9); and gene *Pabpc1* displayed exceptional classification performance (AUC > 0.9).

### 3.12 Validation of differential expression between high- and low-risk groups in dataset GSE193997

**Figure 12:**
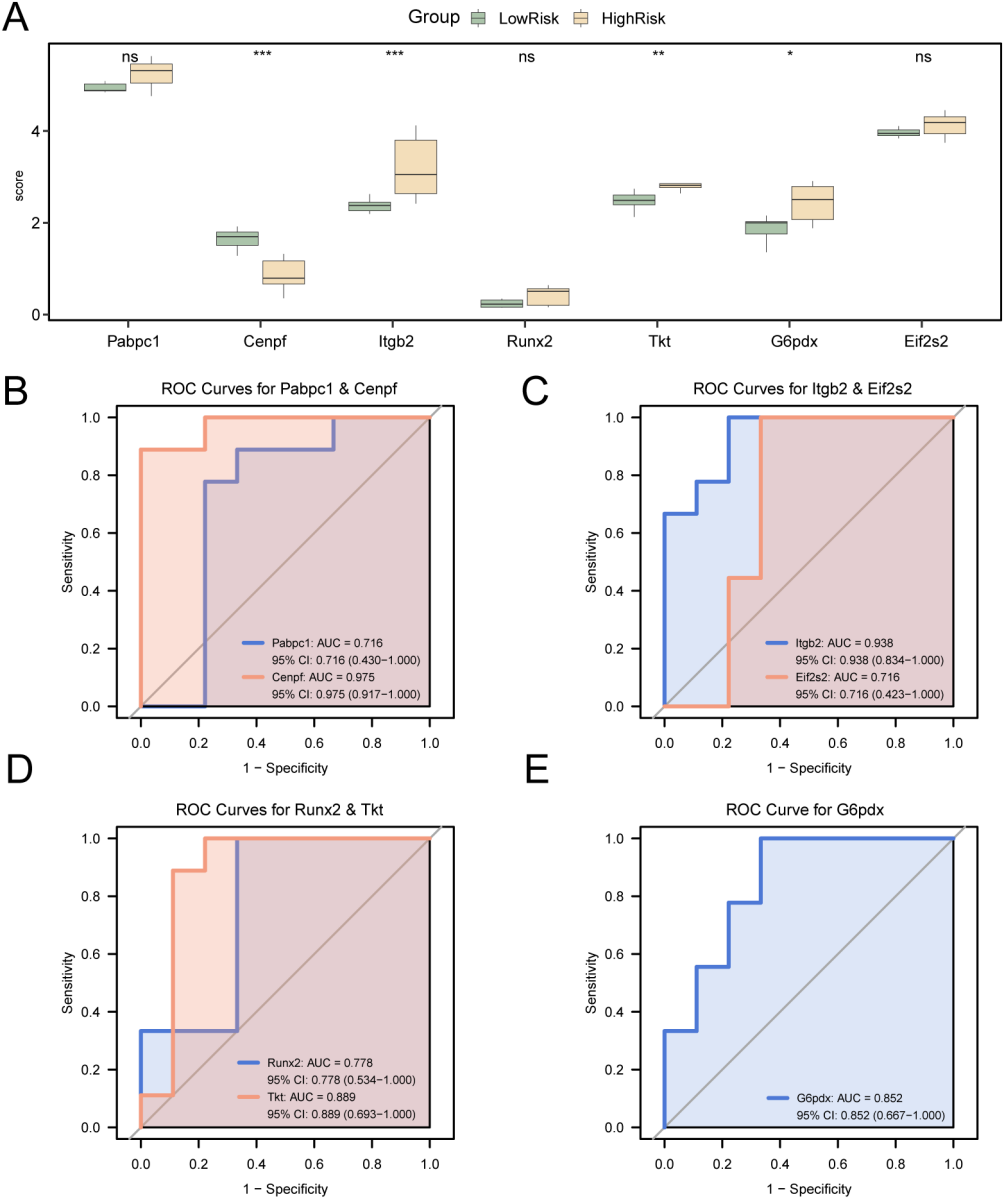
Differential expression validation and ROC curve analysis. **(A)**Group comparison diagram of Key Genes in the dataset GSE193997 cardiac ischemia-reperfusion (MIRI) samples of LowRisk group and HighRisk group. **(B–G)**. Key Genes Pabpc1 and Cenpf **(B)**, Itgb2 and Runx2 **(C)**, Tkt and G6pd **(D)**, ROC curves of Eif2s2 **(E)** in cardiac ischemia-reperfusion (MIRI) samples from Combined GEO Datasets. ROC, Receiver Operating Characteristic; MIRI, Myocardial Ischemic Reperfusion Injury. ns stands for p value ≥ 0.05, no statistical significance; * represents p value < 0.05, statistically significant; ** represents p value < 0.01, highly statistically significant; *** represents p value < 0.001 and highly statistically significant. The closer the AUC is to 1, the better the diagnostic effect is. When AUC > 0.5, it indicates that the expression of the molecule is a trend to promote the occurrence of the event; AUC between 0.7 and 0.9 had a certain accuracy, and AUC above 0.9 had a high accuracy. In the comparison of groups, green represents the LowRisk group and yellow represents the HighRisk group.

Expression levels of the seven model genes (*G6pd, Tkt, Eif2s2, Pabpc1, Itgb2, Cenpf*, and*, Runx2*) were compared between the low- and high-risk groups in the independent MIRI dataset GSE193997 (**Figure 12A**). DEG analysis revealed significant differences (p < 0.05) for all genes, with gene *G6pd* demonstrating the lowest discriminatory capacity among markers.

### 3.13 Myocardial ischemic reperfusion injury subtypes

**Figure 13:**
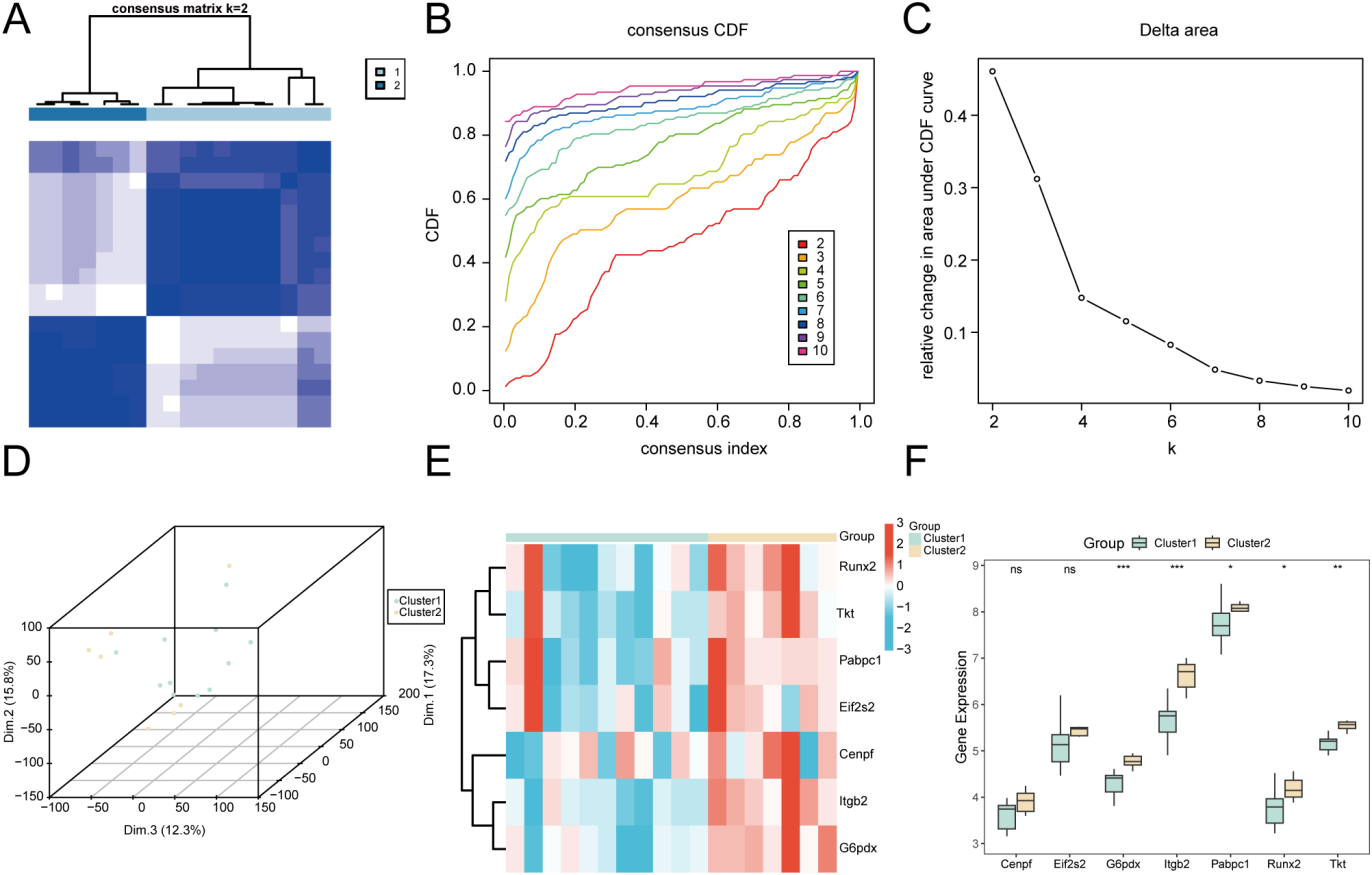
Consensus clustering analysis for MIRI. **(A)** Plot of consensus clustering results for cardiac ischemia-reperfusion (MIRI) samples in the Combined GEO Datasets. **(B, C)**. Consensus CDF, cumulative distribution function plot **(B)** and Delta plot **(C)** of consensus clustering analysis. **(D)** 3D t-SNE cluster map of two disease subtypes of cardiac ischemia-reperfusion (MIRI). **(E)** Heat map of expression values of Key Genes in cardiac ischemia-reperfusion (MIRI) subtypes. **(F)** Comparison map of Key Genes between the two subgroups of cardiac ischemia-reperfusion (MIRI). MIRI, Myocardial Ischemic Reperfusion Injury; CDF, Empirical Cumulative Distribution Function; t-SNE, t-Distributed Stochastic Neighbor Embedding. ns stands for p value ≥ 0.05, not statistically significant; * represents p value < 0.05, statistically significant; ** represents p value < 0.01, highly statistically significant; *** represents p value < 0.001 and highly statistically significant. Green is subtype **(A)** (Cluster1) and yellow is subtype **(B)** (Cluster2). In the heat map of expression values, red is up-regulated and blue is down-regulated.

To identify molecular subgroups within MIRI samples from the integrated GEO dataset, consensus clustering was performed using ConsensusClusterPlus based on the seven key genes’ expression profiles. Two distinct MIRI subgroups were identified: subgroup A (cluster 1, comprising 11 cases) and subgroup B (cluster 2, including 7 cases) (**Figures 13A–C**). The separation of these subgroups was confirmed via t-SNE dimensionality reduction analysis (**Figure 13D**).

Heatmap visualization of key gene expression across subtypes, achieved via pheatmap, revealed distinct patterns (**Figure 13E**). Comparative analysis (**Figure 13F**) validated the differential expression patterns, with several key genes showing significant differences: genes*Pabpc1* and *Runx2* (p < 0.05), *Tkt* (p < 0.01), *Cenpf* and *Itgb2* (p < 0.001).

### 3.14 Gene set variation analysis

**Figure 14:**
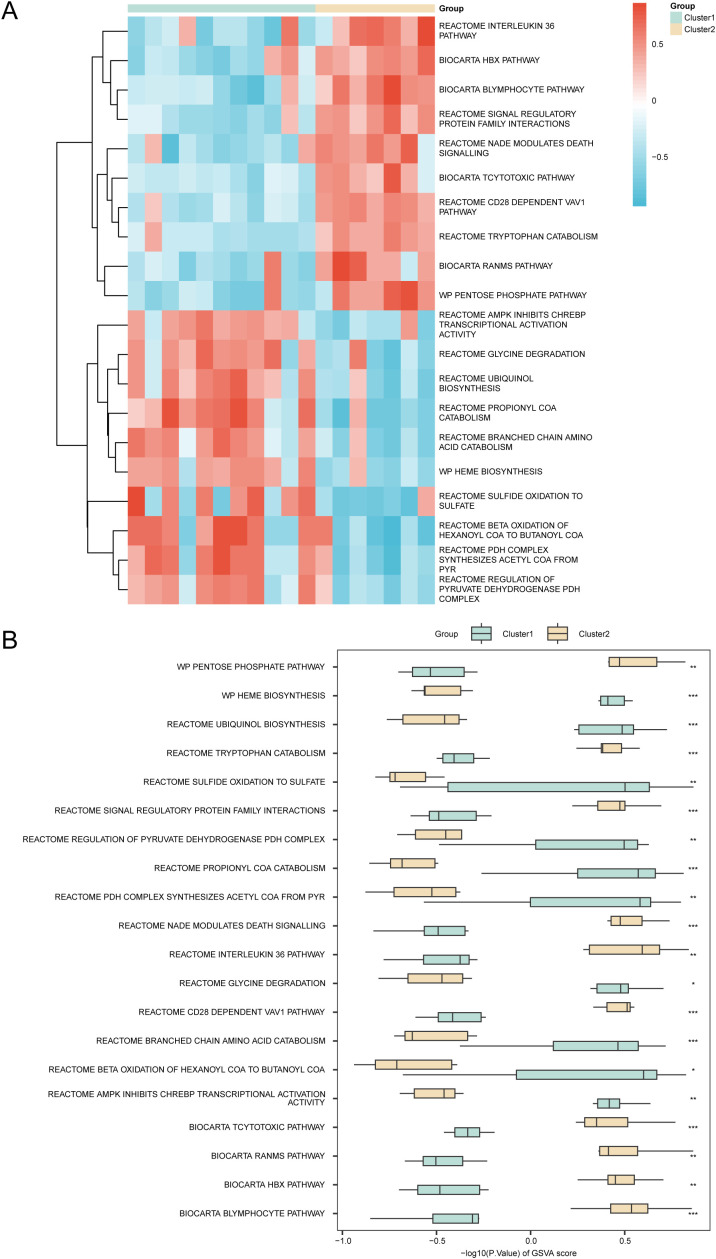
GSVA analysis. **(A, B)** Heat map **(A)** and group comparison map **(B)** of GSVA, gene set variation analysis results among different disease subtypes of cardiac ischemia-reperfusion (MIRI) in Combined GEO Datasets. MIRI, Myocardial Ischemic Reperfusion Injury; GSVA, Gene Set Variation Analysis. * represents p value < 0.05, statistically significant; ** represents p value < 0.01, highly statistically significant; *** represents p value < 0.001 and highly statistically significant. Green represents subtype **(A)** (Cluster1) group and yellow represents subtype **(B)** (Cluster2) group. Blue represents low enrichment and red represents high enrichment in the heat map. The screening criteria for GSVA, gene set variation analysis was adj.p < 0.05, and the p value correction method was BH, Benjamini-Hochberg.

GSVA was applied to assess pathway enrichment differences between MIRI subtypes in the integrated GEO dataset using the MSigDB m2 gene set (v2024.1.Mm.symbols.gmt). Detailed results are provided in **Table 4**. The top 10 positively and negatively enriched pathways (adjusted p < 0.05, ranked by logFC) were visualized in a heatmap showing differential enrichment between subtypes (**Figure 14A**). Mann–Whitney U tests (**Figure 14B**) confirmed statistically significant differences (p < 0.05) in multiple pathways, including the B lymphocyte, HBX, RANMS, cytotoxic T cell, AMPK-mediated CHREBP inhibition, beta-oxidation of hexanoyl-CoA to butanoyl-CoA, branched-chain amino acid catabolism, CD28-dependent VAV1, glycine degradation, interleukin-36, NAD-modulated death signaling, PDH complex acetyl-CoA synthesis, propionyl-CoA catabolism, PDH complex regulation, signal regulatory protein family interaction, sulfide oxidation to sulfate, tryptophan catabolism, ubiquinol biosynthesis, heme biosynthesis, and pentose phosphate pathways.

**Table 4:** Results of GSVA for combined datasets.

ID	logFC	AveExpr	t	P.Value	adj.P.Val	B
BIOCARTA_BLYMPHOCYTE_PATHWAY	0.895316496	-0.01799957	6.229318243	6.97E-07	0.000602216	5.929722577
WP_PENTOSE_PHOSPHATE_PATHWAY	0.871854724	-0.083419726	4.905726418	2.95E-05	0.005665052	2.460269888
REACTOME_SIGNAL_REGULATORY_PROTEIN_FAMILY_INTERACTIONS	0.832717385	-0.064826779	6.001871767	1.32E-06	0.000760255	5.339982854
REACTOME_CD28_DEPENDENT_VAV1_PATHWAY	0.818086804	-0.032973057	6.543858489	2.90E-07	0.0005013	6.737453461
BIOCARTA_HBX_PATHWAY	0.80384465	-0.015184074	5.177080856	1.36E-05	0.003495907	3.17543725
REACTOME_NADE_MODULATES_DEATH_SIGNALLING	0.793405249	-0.05468604	4.785113439	4.15E-05	0.006487004	2.142945878
BIOCARTA_RANMS_PATHWAY	0.787800659	-0.07780817	4.531504032	8.50E-05	0.009345812	1.478165214
REACTOME_INTERLEUKIN_36_PATHWAY	0.773329101	-0.025672131	4.308806845	0.000158969	0.011452368	0.898670308
REACTOME_TRYPTOPHAN_CATABOLISM	0.73682209	-0.032358075	5.745261808	2.72E-06	0.001176298	4.669980092
BIOCARTA_TCYTOTOXIC_PATHWAY	0.707439639	-0.074164324	5.099039439	1.70E-05	0.003680721	2.969673397
WP_HEME_BIOSYNTHESIS	-0.697820199	0.030999849	-4.346081143	0.000143207	0.011452368	0.995309245
REACTOME_AMPK_INHIBITS_CHREBP_TRANSCRIPTIONAL_ACTIVATION_ACTIVITY	-0.698656235	0.035621427	-4.207318317	0.000211086	0.012335374	0.636394375
REACTOME_REGULATION_OF_PYRUVATE_DEHYDROGENASE_PDH_COMPLEX	-0.699857914	0.008633863	-4.163465257	0.000238514	0.012887207	0.523481814
REACTOME_GLYCINE_DEGRADATION	-0.725484068	0.057288374	-3.831787064	0.000595689	0.021095652	-0.320610228
REACTOME_SULFIDE_OXIDATION_TO_SULFATE	-0.75139999	-0.081905103	-3.351552128	0.002161792	0.035606087	-1.500856824
REACTOME_BRANCHED_CHAIN_AMINO_ACID_CATABOLISM	-0.764941523	0.02500467	-4.525471473	8.65E-05	0.009345812	1.462406541
REACTOME_UBIQUINOL_BIOSYNTHESIS	-0.776076304	0.028570348	-4.669615081	5.76E-05	0.007656724	1.839694539
REACTOME_PDH_COMPLEX_SYNTHESIZES_ACETYL_COA_FROM_PYR	-0.801784594	0.015053703	-4.127935419	0.000263283	0.013312024	0.432198906
REACTOME_BETA_OXIDATION_OF_HEXANOYL_COA_TO_BUTANOYL_COA	-0.813516793	-0.010932789	-3.616512758	0.001068202	0.025253131	-0.856880327
REACTOME_PROPIONYL_COA_CATABOLISM	-0.938696101	0.042416041	-5.164160588	1.42E-05	0.003495907	3.14137131

GSVA, Gene Set Variation Analysis.

### 3.15 Immune infiltration analysis of myocardial ischemic reperfusion injury via CIBERSORT

**Figure 15:**
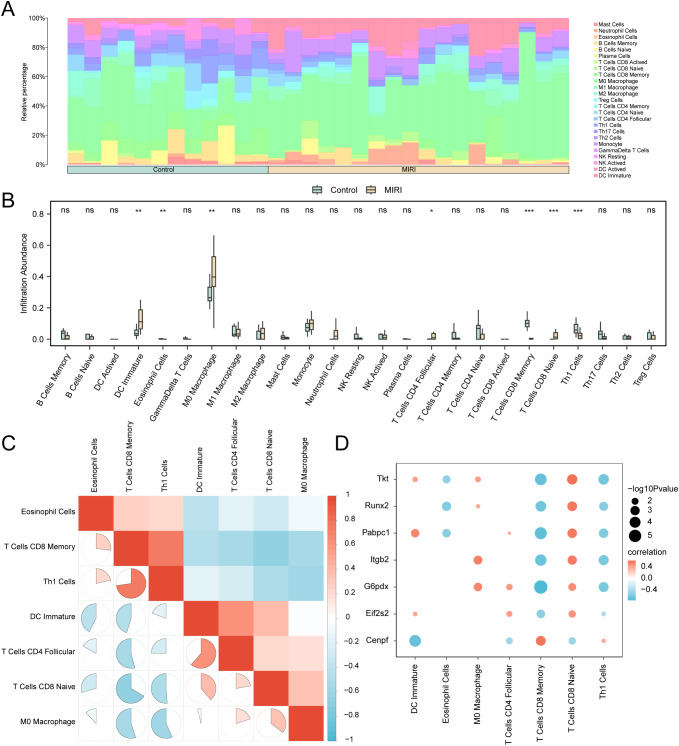
Combined datasets immune infiltration analysis by CIBERSORT algorithm. **(A, B)** The proportion of immune cells in the integrated GEO Datasets (Combined Datasets) bar graph **(A)** and group comparison graph **(B)**. **(C)** Correlation heatmap of immune cells in the integrated GEO Datasets (Combined Datasets). **(D)** Bubble plot of the correlation of Key Genes with immune cell infiltration abundance in the Combined GEO Datasets. ns stands for p value ≥ 0.05, not statistically significant; * represents p value < 0.05, statistically significant; ** represents p value < 0.01, highly statistically significant; *** represents p value < 0.001 and highly statistically significant. MIRI, Myocardial Ischemic Reperfusion Injury. The absolute value of correlation coefficient (r value) below 0.3 was weak or no correlation, between 0.3 and 0.5 was weak correlation, between 0.5 and 0.8 was moderate correlation, and above 0.8 was strong correlation. Green is the Control group, yellow is the myocardial ischemia-reperfusion (MIRI) group. Red shows positive correlation, blue shows negative correlation. The depth of the color represents the strength of the correlation.

CIBERSORT was used to assess immune cell infiltration patterns across 25 immune cell types in the integrated GEO dataset. Stacked bar plots depicted compositional differences in these immune cell subsets (**Figure 15A**). Comparative analysis between experimental and control groups (**Figure 15B**) identified seven immunocyte populations with significantly differential infiltration (p < 0.05), including eosinophils, naïve and memory CD8**^+^** T lymphocytes, M0 macrophages, follicular helper CD4**^+^** T cells, Th1 cells, and immature dendritic cells. Spearman correlation analysis, presented as a heatmap (**Figure 15C**), revealed robust interactions among immune cell types, including an inverse relationship between naïve and memory CD8**^+^** T cells (r = −0.665, p < 0.05) and a positive association between memory CD8**^+^** T cells and Th1 lymphocytes (r = 0.728, p < 0.05). Gene–immune cell correlations, visualized in bubble plots (**Figure 15D**), showed that gene *G6pd* was negatively correlated with memory CD8**^+^** T cells (r = −0.758, p < 0.05) and gene *Tkt* positively correlated with naïve CD8**^+^** T cells (r = 0.617, p < 0.05).

### 3.16 Immune infiltration analysis of myocardial ischemic reperfusion injury subtypes via CIBERSORT

**Figure 16:**
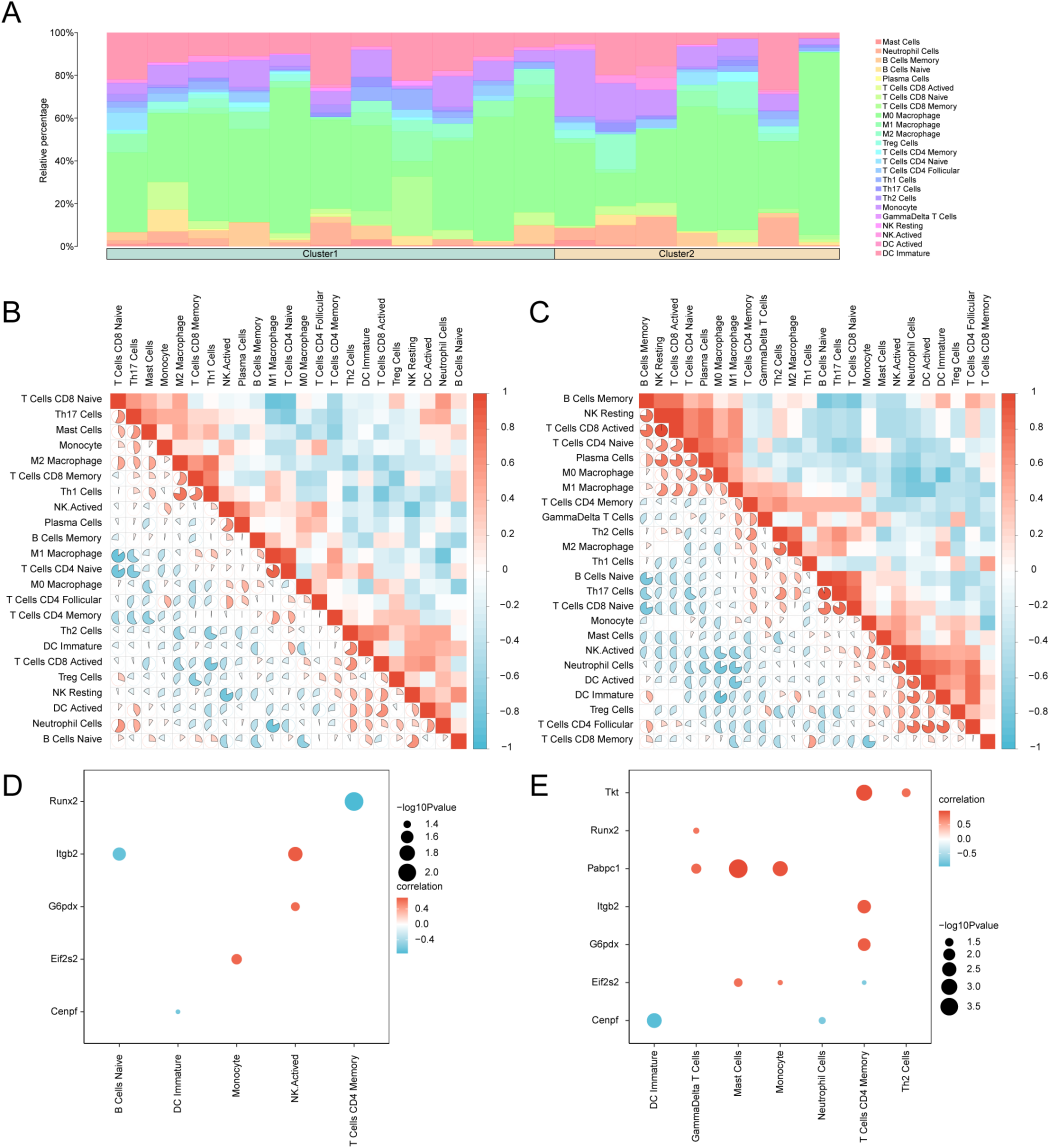
Cluster group immune infiltration analysis by CIBERSORT algorithm. **(A)** Bar chart of the proportion of immune cells in subtype **(A)** (Cluster1) and subtype **(B)** (Cluster2) of cardiac ischemia-reperfusion (MIRI) samples. B-C. The results of correlation analysis of immune cell infiltration abundance in subtype **(A)** (Cluster1) **(B)** and subtype **(B)** (Cluster2) **(C)** of cardiac ischemia-reperfusion (MIRI) samples are shown. **(D, E)**. Bubble plot of correlation between immune cell infiltration abundance and Key Genes in subtype A (Cluster1) **(D)** and subtype B (Cluster2) **(E)** of cardiac ischemia-reperfusion (MIRI) samples. MIRI, Myocardial Ischemic Reperfusion Injury. The absolute value of correlation coefficient (r value) below 0.3 was weak or no correlation, between 0.3 and 0.5 was weak correlation, between 0.5 and 0.8 was moderate correlation, and above 0.8 was strong correlation. Green is subtype A (Cluster1), yellow is subtype B (Cluster2). Positive correlations are shown in red and negative ones in blue. The depth of the color represents the strength of the correlation.

CIBERSORT was again used to quantify immune cell proportions across 25 distinct immune cell subtypes in the integrated GEO cohort. Stacked bar plots illustrated subtype-specific distributions (**Figure 16A**), and subsequent analysis revealed differential immune cell interaction networks across the subtypes. In Cluster 1 (subtype A), naïve CD8^+^ T lymphocytes were inversely correlated with M1 macrophages (Pearson’s r = −0.856, p < 0.05), whereas M1 macrophages were positively correlated with naïve CD4^+^ T cells (r = 0.823, p < 0.05) (**Figure 16B**). In Cluster 2 (subtype B), M0 macrophages were inversely correlated with immature dendritic cells (r = −0.857, p < 0.05), whereas activated CD8^+^ T cells correlated perfectly with resting natural killer cells (r = 1.00, p < 0.05) (**Figure 16C**).

Correlation bubble plots visualized distinct gene–immune cell relationships across subtypes. In subtype A, gene *Runx2* was negatively correlated with memory CD4^+^ T cells (r = −0.748, p < 0.05), whereas gene *Itgb2* was correlated positively with activated natural killer cells (r = 0.689, p < 0.05) (**Figure 16D**). In subtype B, gene *Cenpf* was negatively correlated with immature dendritic cells (r = −0.932, p < 0.05), whereas gene *Pabpc1* was positively correlated with mast cells (r = 0.978, p < 0.05) (**Figure 16E**).

### 3.17 Expression of key genes in HL-1 cardiomyocytes

**Figure 17:**
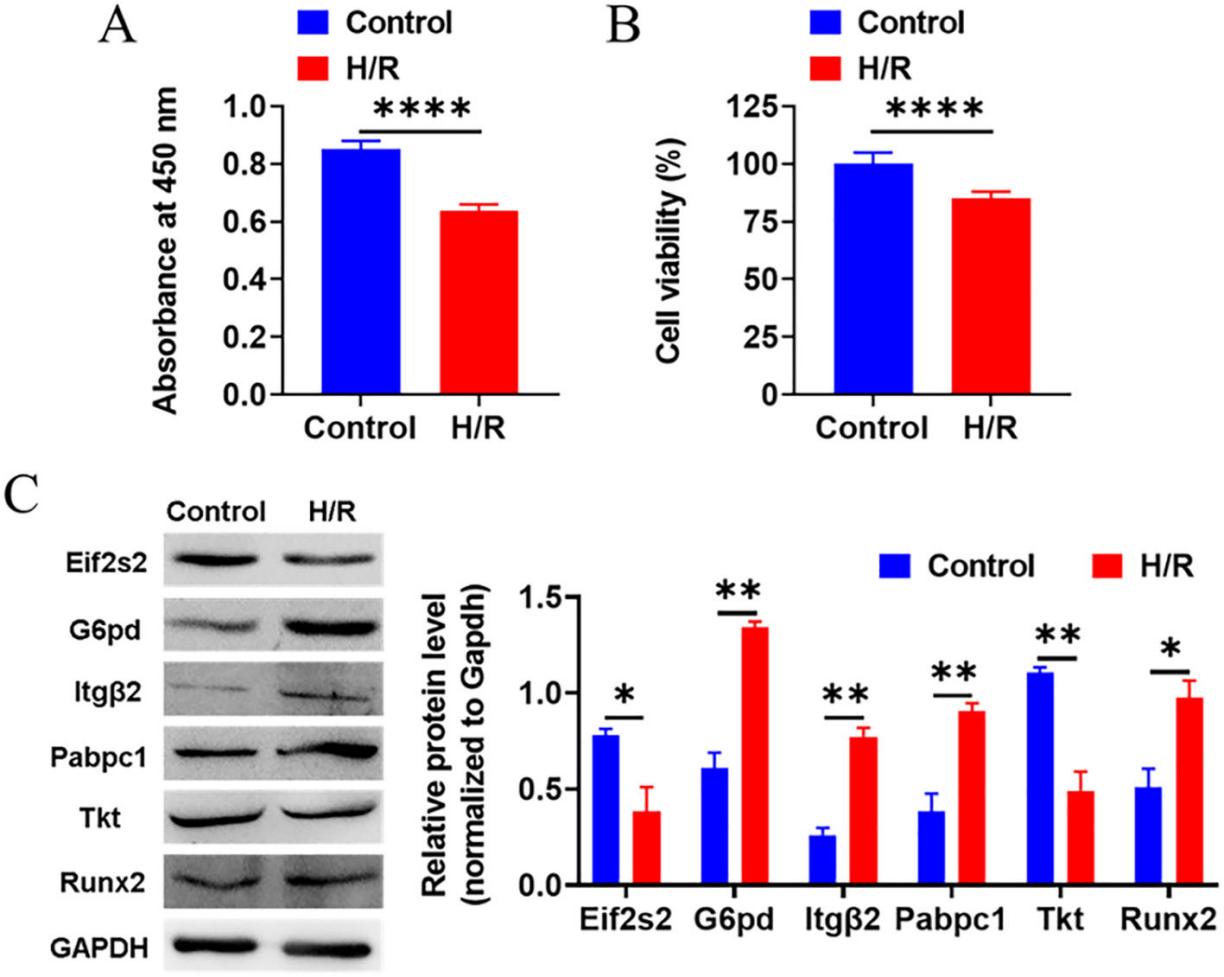
Validation of the expression of the hub genes screened by the bioinformatics analysis using Western blotting in H/R-treated cardiomyocytes. HL-1 cardiomyocytes were divided into Control (cultured in 5%CO2-21%O2 atmosphere) and Hypoxia-reoxygenation (H/R, cultured in 5%CO2-3%O2 for 4 h and then in 5%CO2-21%O2 atmosphere for 24 h) groups. Cell viability was detected with CCK-8 assay. **(A)** Absorbance at 450 nm. **(B)** Percentages of Cell viability normalized to Control. **(C)** Western blotting was used to validate the expression of the hub genes screened by the bioinformatics analysis. *P<0.05, **P<0.01, ****P<0.0001.

Western blot analysis revealed differential expression of the seven candidate genes in HL-1 cardiomyocytes. Compared with controls, the MIRI group exhibited significant upregulation of genes *G6pd*, *Itgb2, Pabpc1* (p < 0.01), and *Runx2* (p < 0.05). Conversely, genes *Eif2s2* (p < 0.05) and *Tkt* (p < 0.01) were significantly downregulated in the MIRI group. Notably, gene *Cenpf* expression was undetectable in the H/R experimental group (**Figure 17**).

## 4 Discussion

MIRI remains a major clinical challenge, causing substantial morbidity and mortality despite advances in reperfusion therapy (37, 38) and imposing a heavy burden on healthcare systems (39). Inspired by prior studies on metabolic reprogramming in MIRI and the emerging role of lactylation as a histone mark that directs transcriptional outputs (Zhang et al) (40)., we hypothesized that lactylation-mediated epigenetic regulation may be involved in the mechanism underlying MIRI. During MIRI, lactate accumulation provides a biochemical basis for protein lactylation. Previous studies have indicated that lactylation can be involved in processes such as inflammatory regulation and metabolic reprogramming (40). In this study, we integrated GEO datasets using differential gene expression analysis, WGCNA, and LRGs from GeneCards, combined with machine learning techniques. Our integrated approach identified seven key LRGs (*G6pd, Tkt, Eif2s2, Pabpc1, Itgb2, Cenpf*, and *Runx2*) strongly associated with MIRI. Functional analyses revealed that these genes participate in essential biological processes, including metabolic regulation via the pentose phosphate pathway (mediated by genes *G6pd* and *Tkt*), immune regulation (with gene *Itgb2* facilitating leukocyte adhesion), and stress response modulation (with gene *Eif2s2* influencing protein synthesis). These findings underscore the potential of these genes to serve as early diagnostic biomarkers and therapeutic targets for MIRI. However, the present study focused on the expression level of LRGs and their association with MIRI, without directly examining the lactylation modification status of the corresponding proteins in myocardial tissues or cellular models. Thus, the precise role of lactylation in immune regulation and signal transduction would need to be further elucidated through protein modification detection and functional experiments.

Among the key genes, *G6pd* and *Tkt* encode glucose-6-phosphate dehydrogenase and transketolase, respectively, both rate-limiting enzymes in the pentose phosphate pathway (41, 42). These enzymes enhance pentose phosphate pathway flux to maintain metabolic and redox homeostasis (43). Specifically, NADPH produced by *G6pd* supports glutathione regeneration and reactive oxygen species scavenging, whereas *Tkt* links glycolysis to nucleotide synthesis. This metabolic adaptation appears to be fine-tuned by lactylation, a regulatory mechanism identified here. Specifically, lactylation potentiate G6pd activity, a phenomenon also observed in cancer cell lines by Zhang Y et al. (44). Concurrently, Eif2s2 and Pabpc1 might cooperatively maintain proteostasis under reperfusion stress: Eif2s2 (45) suppresses global translation, while Pabpc1 (46) stabilizes stress-responsive transcripts, collectively influencing cardiomyocyte survival. Gene *Itgb2* contributes to inflammatory injury via leukocyte adhesion and infiltration, consistent with the work of Yuan Z et al. (47), where *Itgb2* inhibition attenuated myocardial damage. Overall, these genes influence MIRI pathogenesis through interconnected mechanisms involving redox regulation (*G6pd* and *Tkt*), translational control (*Pabpc1*), and inflammation (*Itgb2*). Their combined functions underscore the complex nature of MIRI and highlight promising directions for future diagnostic and therapeutic strategies.

GO analysis revealed LRDEG enrichment in nuclear chromosome segregation and cytoskeletal reorganization, suggesting that lactylation modulates cardiomyocyte structural integrity and stress adaptation (40). KEGG pathway analysis identified key signaling pathways, including TLR4/NF-κB–mediated inflammation (48), FcγR-mediated phagocytosis (49), tight junction disruption (50), and cancer-like metabolic reprogramming (51). These findings implicate lactylation as an important regulator of inflammation, cytoskeletal dynamics, and metabolic adaptation in MIRI, supporting its potential as a therapeutic target. In the pathological context of MIRI, inflammatory cell recruitment and proliferation, fibroblast activation, and tissue remodeling all involve cell cycle regulation and structural reorganization. During ischemia reperfusion, oxidative stress and energy metabolism disturbance can lead to alterations in the cytoskeletal structure of cardiomyocytes, affecting cellular stability and contractile function. Meanwhile, lactate accumulation not only reflects a state of metabolic stress but is also considered to function as a signaling molecule that participates in inflammatory regulation and metabolic reprogramming (52). Therefore, the enrichment results may suggest that, under metabolic stress, networks related to cell proliferation and structural remodeling undergo reorganization, though the precise molecular mechanisms still require further validation.This series of processes highlights the urgency and complexity of translating basic research findings into clinically effective therapies. Future cardioprotective strategies need to integrate a deep understanding of metabolic, inflammatory, and structural remodeling pathways and explore personalized intervention timing and targets for these processes (53).

GSEA results highlighted a coherent pathological cascade in MIRI. Specifically, acute sterile inflammation, indicated by the enrichment of inflammatory response genes and neutrophil degranulation, initiates tissue injury. This inflammatory environment triggers an epithelial-to-mesenchymal transition–like response in fibroblasts, priming them for excessive collagen production. The cascade culminates in fibrotic remodeling, evidenced by collagen fibril assembly enrichment, reflecting a maladaptive repair process evolving from acute inflammation to chronic structural dysfunction. These findings align with established MIRI mechanisms and emphasize the importance of transcriptomic profiling in understanding disease progression (54, 55).

WGCNA identified three modules strongly associated with MIRI. Intersection with LRGs revealed 33 core “module-lactylation” genes, highlighting a novel network in which lactate accumulation during ischemic stress induces protein lactylation, modulating gene networks associated with MIRI (47). Lactylation of these genes likely influences metabolic adaptation, inflammatory responses, and fibrotic signaling, positioning protein lactylation as a key “metabolic–epigenetic” regulator in MIRI pathogenesis.

GSVA revealed enrichment of immune regulatory pathways, including B lymphocyte, cytotoxic T cell, and CD28-dependent VAV1 signaling, as well as metabolic pathways, such as AMPK-mediated ChREBP inhibition and the pentose phosphate pathway. These pathways intersect functionally with diagnostic genes *G6pd, Tkt, Eif2s2, Pabpc1, Itgb2, Cenpf*, and *Runx2*, which play central roles in MIRI pathology: *G6pd* and *Tkt* maintain NADPH through the pentose phosphate pathway, regulating oxidative stress; *Eif2s2* and *Pabpc1* stabilize inflammation-related mRNAs, amplifying cytokine release; *Itgb2* promotes FcγR-mediated phagocytosis and neutrophil degranulation, exacerbating inflammation; *Cenpf* disrupts cell cycle regulation, inducing cardiomyocyte death; and *Runx2* drives fibroblast activation and extracellular matrix deposition, promoting fibrosis. Together, these gene–pathway interactions form an integrated network that amplifies immune activation, metabolic dysregulation, and oxidative stress in MIRI, highlighting promising therapeutic targets (49, 51, 56, 57).

Our MIRI diagnostic model showed strong discriminative ability during internal validation (AUC > 0.9) but limited generalizability across external cohorts (AUC = 0.5–0.7). This discrepancy indicates potential overfitting driven by dataset heterogeneity, including technical and population differences. Among the biomarkers, *Itgb2* emerged as a highly specific diagnostic marker, with its expression levels correlating with inflammatory severity. As a leukocyte-specific adhesion molecule, *Itgb2* protein promotes neutrophil and monocyte infiltration into ischemic myocardium and activates NF-κB–mediated proinflammatory cytokine release, underscoring its central role in MIRI-related inflammation (56). In external validation, *G6pd* also maintained significant predictive value (p < 0.05), aligning with its role in sustaining NADPH-dependent redox homeostasis under oxidative stress (58). Variability in the performance of other genes reflects the challenges of translating transcriptomic signatures into clinical diagnostics and highlights the need for multi-omics integration and advanced algorithms, such as ensemble learning, to improve robustness and applicability.

Our findings highlight a major immunometabolic shift in MIRI, where alterations in specific metabolic pathways are strongly associated with shifts in immune cell populations, suggesting a potential functional influence. For instance, we discovered a strong inverse relationship between *G6pd* and memory CD8^+^ T cells (r =−0.758). This aligns with existing models suggesting that metabolic activity plays a key role in determining T-cell differentiation paths (59). We also noted coordinated changes among T-cell subsets, such as the negative correlation between naïve and memory CD8^+^ T cells (r = −0.665), echoing earlier reports of immune repopulation following ischemic damage (60). Importantly, our work goes a step further by quantitatively tying these immune shifts to metabolic alterations, pointing to a possible mechanistic link between immunometabolism and immune cell makeup—an idea that fits with the growing recognition of metabolism as a regulator of immune function (61). Focusing on immune heterogeneity (62), our cluster-based analysis uncovered unique immunometabolic signatures. In one subgroup, a striking inverse correlation emerged between the cell cycle-related gene *Cenpf* and immature dendritic cells (r = −0.932). This previously unnoticed connection hints that cell cycle processes might help shape innate immune responses in MIRI, raises new questions for future research. It should be noted that different immune cell deconvolution algorithms vary in the selection of feature gene sets and statistical modeling frameworks, which may have a certain impact on the estimation results of immune cell proportions. In this study, the CIBERSORT method was used to analyze the relative changes in immune cell composition, with a focus on the differences in trends between groups and their correlations with the expression of key genes. The relevant results reflect the overall characteristics of the MIRI immune microenvironment, but it is still necessary to further confirm them in the future by combining other algorithms or experimental verification methods.

There are significant differences in the infiltration levels of neutrophils and macrophages among different MIRI subtypes. By combining the expression changes of key lactylation-related genes among subtypes and the correlation analysis results with the abundance of immune cell infiltration, it can be observed that the expression trends of some genes are associated with the degree of inflammatory cell infiltration to a certain extent. Given the core role of neutrophils and macrophages in the early inflammatory response, tissue damage, and subsequent repair processes during ischemia-reperfusion, the above results suggest that lactylation-related genes may be involved in the regulation of the immune microenvironment or the inflammatory process. However, this association is currently based on expression correlation analysis, and the specific regulatory mechanism still needs to be verified by further experimental studies.

Western blot validation in HL-1 cardiomyocytes subjected to H/R confirmed significantly elevated *G6pd, Itgb2, Pabpc1*, and *Runx2* protein levels (p < 0.05), consistent with transcriptomic upregulation observed in GEO datasets, such as GSE108940. These results reinforce the translational-level concordance for *G6pd*, *Itgb2*, *Pabpc1*, and *Runx2*, supporting their roles as strong biomarker candidates in MIRI. Conversely, *Tkt* and *Eif2s2* proteins showed significantly reduced expression despite increased mRNA levels, even after GAPDH normalization. The observed transcript-protein discordance for Tkt and Eif2s2 points directly to potent post-transcriptional regulation (e.g., translational inhibition or protein degradation) during MIRI. This finding not only unveils a new layer of regulatory complexity but also provides an experimental foundation for mechanistic studies, clinical development, and efficient resource allocation.

Although to our knowledge this study is the first to examine LRGs in MIRI through a bioinformatics framework, several limitations must be acknowledged. First, all results derive from mouse transcriptome data, and key diagnostic genes have not been validated in human MIRI tissues, limiting translational relevance. Second, several GEO datasets, including GSE193997, contain small sample sizes (e.g., only three controls), reducing statistical power and generalizability. Third, functional insights rely primarily on bioinformatics-based predictions and *in vitro* validation using an HL-1 cardiomyocyte H/R model. Future research should integrate *in vivo* models, such as MIRI mouse knockouts, and clinical specimens to verify lactylation-dependent regulatory mechanisms. In addition, the present study is primarily based on mouse transcriptomic data and *in vitro* H/R models for analysis and validation, and the expression levels or lactylation modification status of the relevant genes have not yet been evaluated in human MIRI samples. Due to species differences and the complexity of clinical pathology, further validation of their expression profiles in human myocardial tissues or peripheral blood samples is crucial for assessing their potential clinical application value. Furthermore, conducting correlation analyses between gene or protein expression levels and clinical parameters-such as infarct size, postoperative cardiac function changes, and inflammatory indicators-would help clarify their feasibility as potential diagnostic or risk-stratification biomarkers. These issues warrant further investigation in future clinical studies.

In conclusion, this study reinforces established MIRI pathways while identifying protein lactylation as a key “metabolic–epigenetic” regulator of injury-driven gene expression. The seven identified LRGs provide insights into the metabolic and inflammatory mechanisms underlying MIRI and provide a foundation for future diagnostic and therapeutic development. Translation into clinical practice will require validation in human samples and more in-depth mechanistic investigations of lactylation-mediated regulation. It is important to note that our study, while implicating lactylation through gene expression associations and protein-level validation, did not directly assess the lactylation modification status of the corresponding proteins in myocardial tissues or models. The specific changes in protein lactylation levels and their modification sites remain to be experimentally verified. Future research should aim to directly confirm and characterize these modifications. This could be achieved by employing pan-Kla antibody immunoprecipitation or mass spectrometry to profile lactylation in MIRI. Subsequent site-specific mapping, coupled with functional assays, will be crucial to clarify the impact of lactylation on protein function—such as enzymatic activity, stability, or localization—and thereby fully elucidate its precise molecular regulatory role in MIRI pathogenesis.

## Data Availability

The original contributions presented in the study are included in the article/**Supplementary Material**. Further inquiries can be directed to the corresponding author.
